# Supplementary material for: Environmental metagenomics enhances detection of circulating viruses from live poultry markets in Cambodia
Source: Nat Commun. 2026 Jan 12;17:1525. doi: 10.1038/s41467-025-68245-8 (PMC12891645; doi:10.1038/s41467-025-68245-8)
Supplement: Supplementary file 1 — Supplementary Information [file 41467_2025_68245_MOESM1_ESM.pdf]

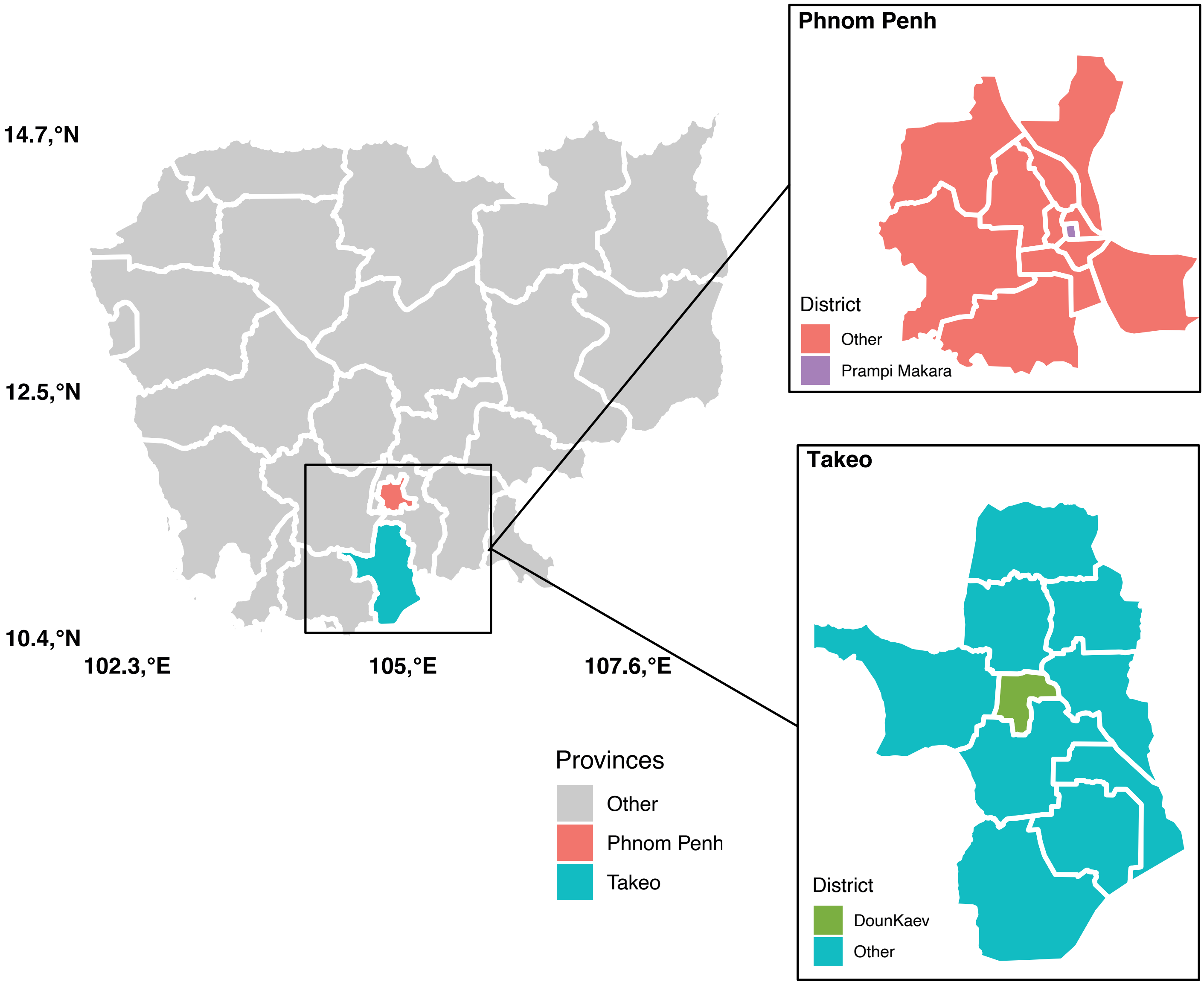

Supplementary Figure 1: Geographic location of live-bird makrets sampled in Cambodia. The main panel shows a map of Cambodia highlighting the two provinces where environmental surveillance was conducted: Phnom Penh and Takeo. Insets provide detailed maps of each province, showing the specific districts where live bird markets were located. In Phnom Penh, Orussey Market is situated in the central district of Prampi Makara. In Takeo, Doun Keo Market is located in the district of Doun Kaev.

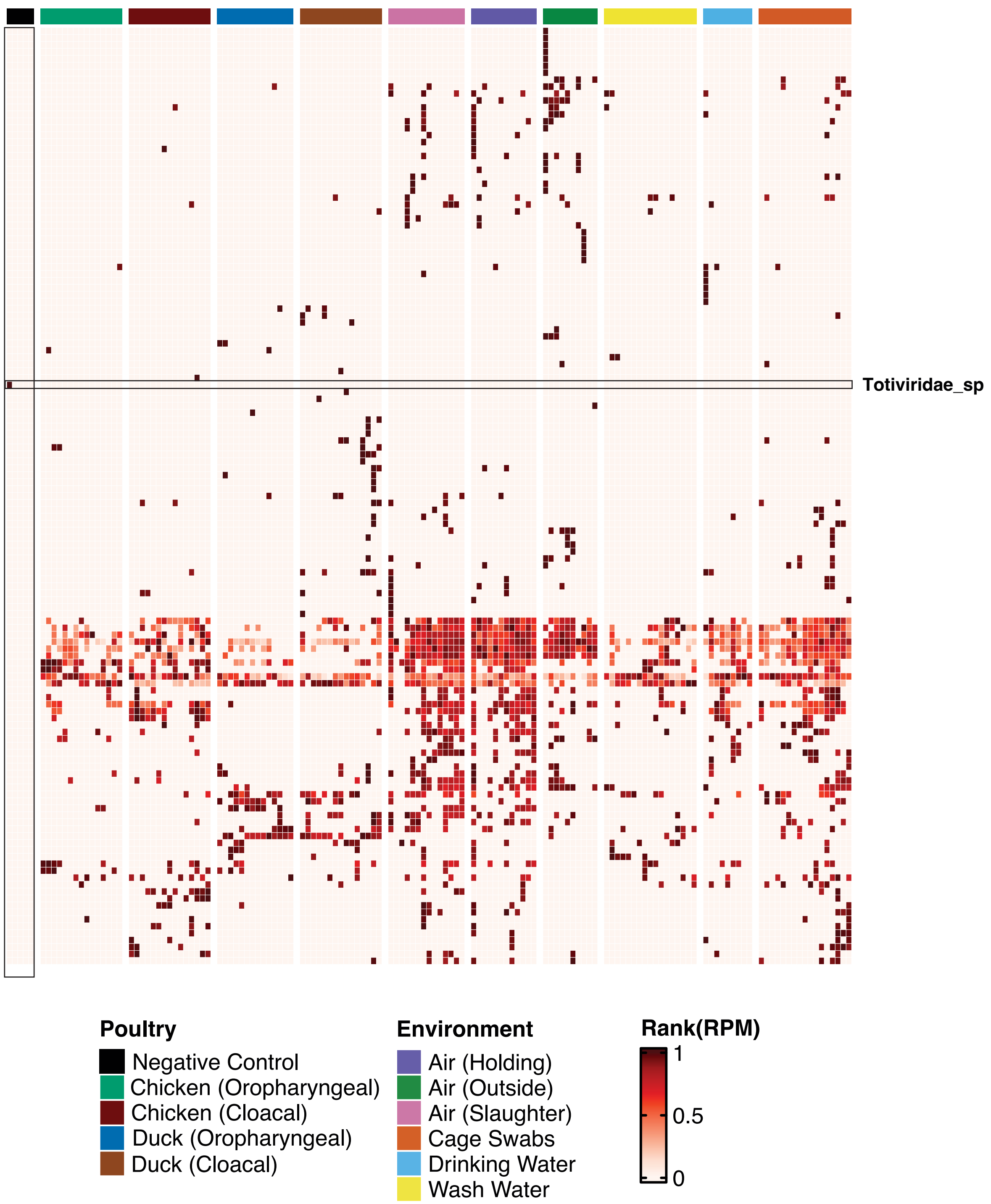

Supplementary Figure 2. Sequenced negative controls show no evidence of poultry virus contamination. Heatmap showing ranked abundances (RPM) of all viruses detected in this study. Columns represent individual samples clustered by group, including five negative controls for comparison. Rows represent viruses; for clarity, only taxa detected in the negative controls are labeled. A black vertical square highlights that no other viral taxa were detected in the negative controls except for an unclassified Totiviridae. A black horizontal square indicates that this Totiviridae was not detected in any poultry or environmental samples collected from live bird markets (LBMs) in Cambodia.

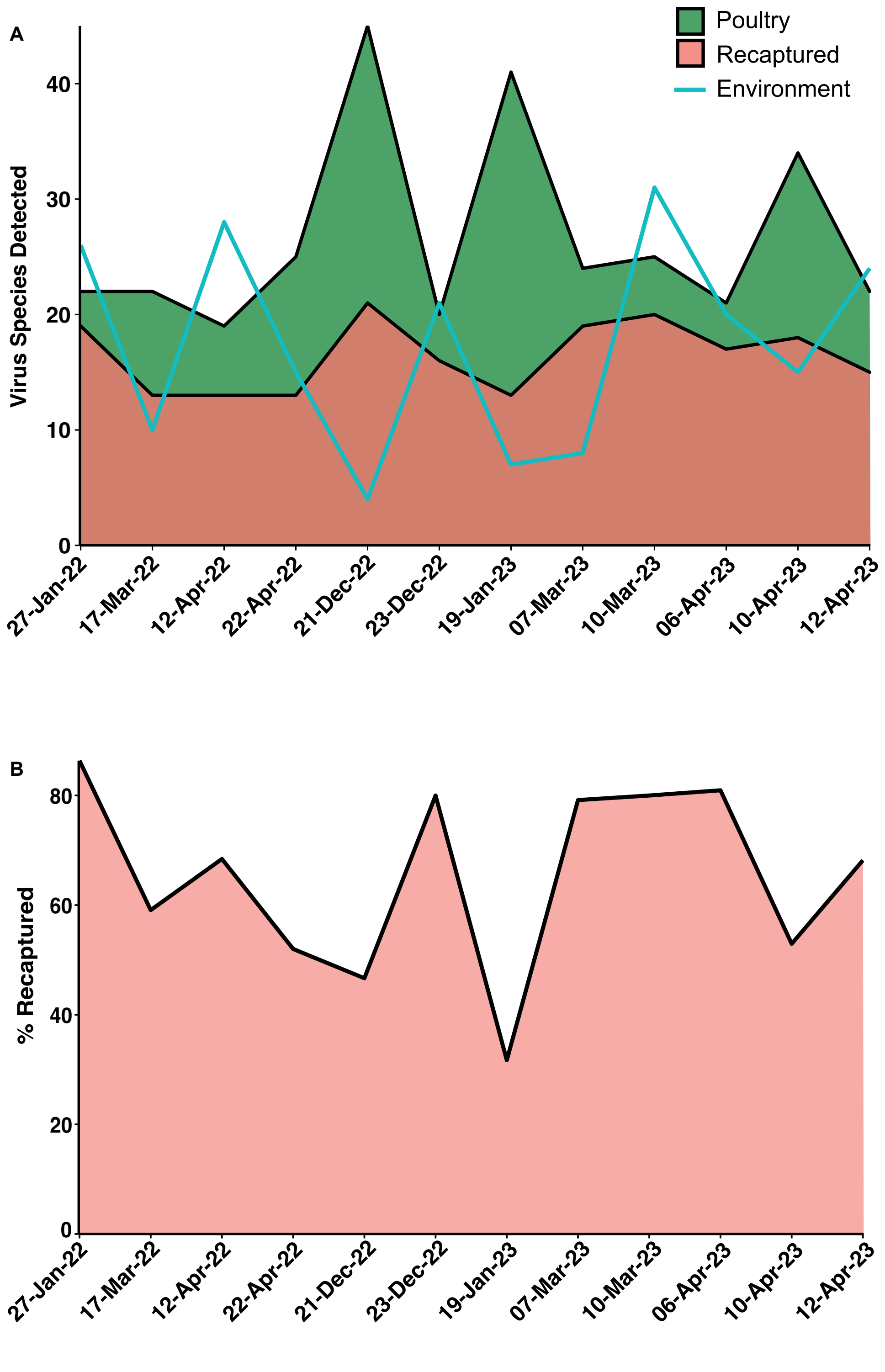

Supplementary Figure 3. Environmental sampling recaptures between 70% and 90% of viruses detected in poultry. (A) The total number of virus species detected in poultry swabs and the subset of those also detected in environmental samples at each timepoint. The blue line indicates poultry viruses that were uniquely recaptured by environmental samples at each timepoint. (B) The data from (A) expressed as the percentage of poultry-detected viruses that were also recaptured by environmental sampling.

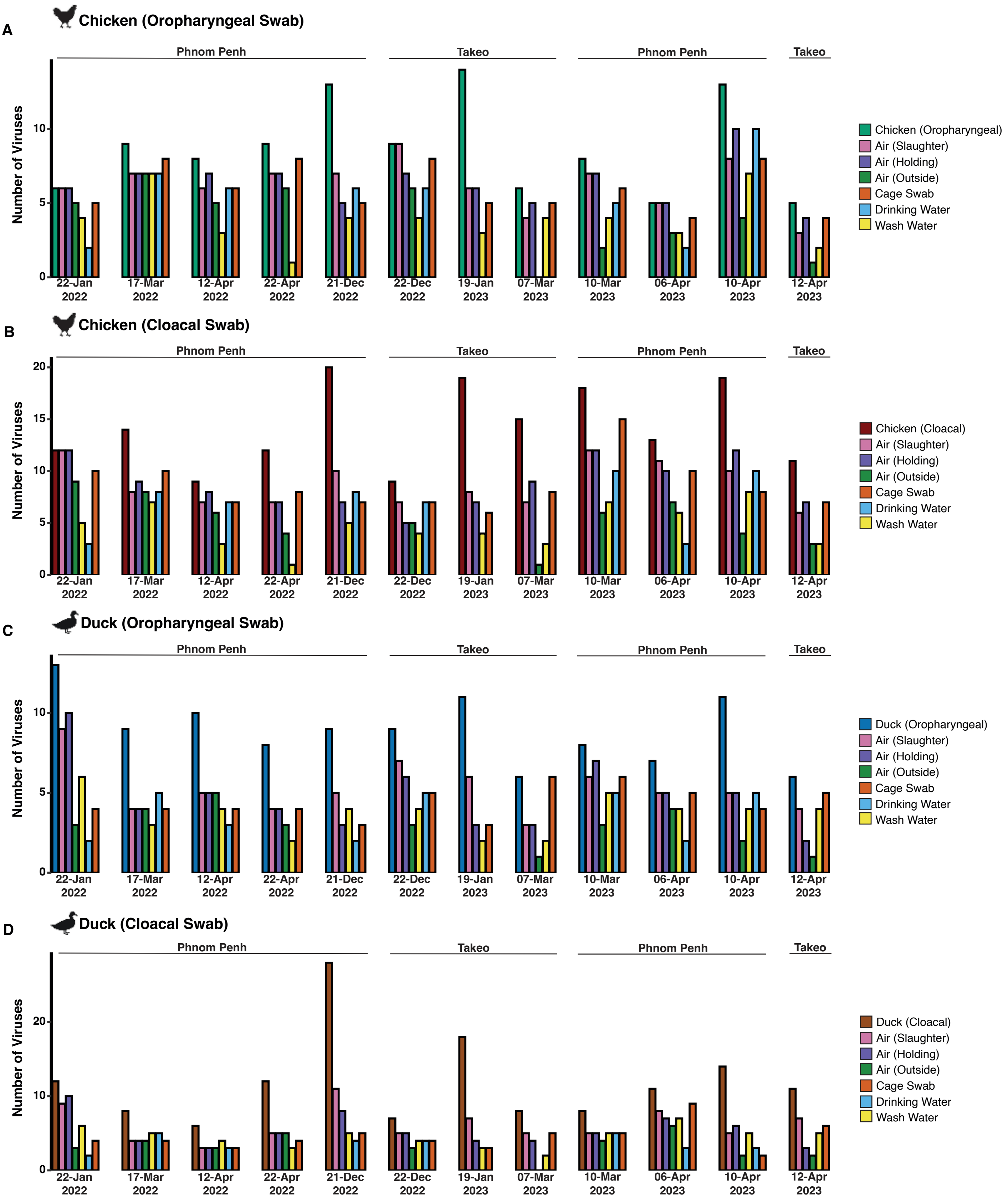

Supplementary Figure 4. Individual environmental samples recapture the majority of viruses detected in different poultry swab types.. Barplots showing the number of viruses detected in A) chicken oropharyngeal, B) chicken cloacal, C) duck oropharyngeal and D) duck cloacal swabs compared to the environmental samples at each timepoint. For each environmental sample type, the totals only include viruses that were identified in the respective poultry swab type at that specific timepoint. Thus, the data shown focuses solely on the ability of environmental samples to recapture what was found in poultry swabs and does not include additional poultry viruses detected at that time.

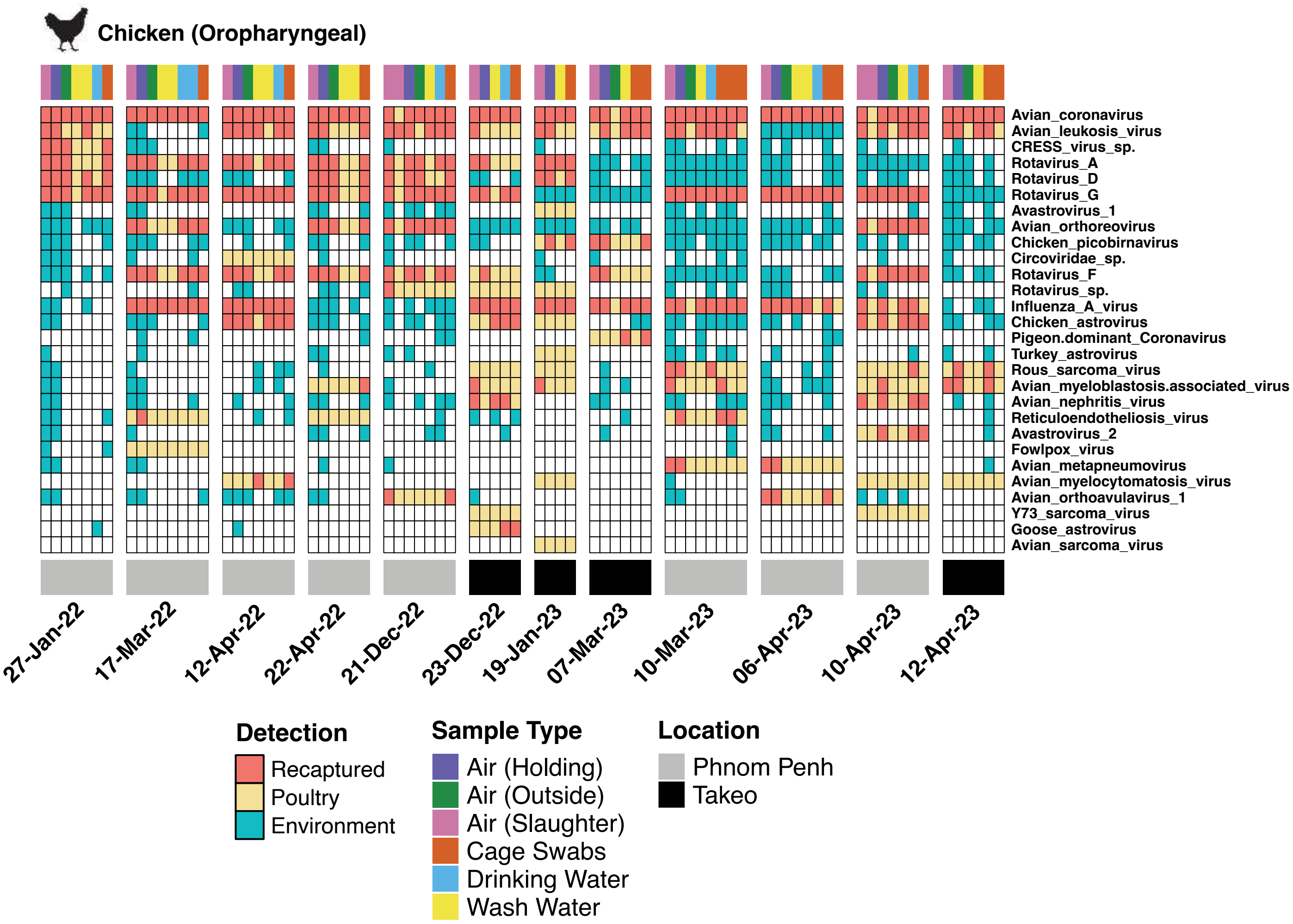

Supplementary Figure 5. Detection of chicken oropharyngeal viruses through environmental sampling. Heatmap showing 28 different virus species which were detected at least once in chicken oropharyngeal swabs over the course of the study. The red color shows when a virus was identified in chicken oropharyngeal swabs and at the exact same timepoint was recaptured in the environment. The pale yellow colour shows when a virus species was identified in chicken oropharyngeal swabs but failed to be recaptured through environmental sampling. The blue colour shows virus species which were found in the environment but failed to be detected in chicken oropharyngeal swabs. Rows of the heatmap represent virus species and columns represent individual environmental samples (annotated at the top of the heatmap). The location of each timepoint is annotated on the bottom of the heatmap.

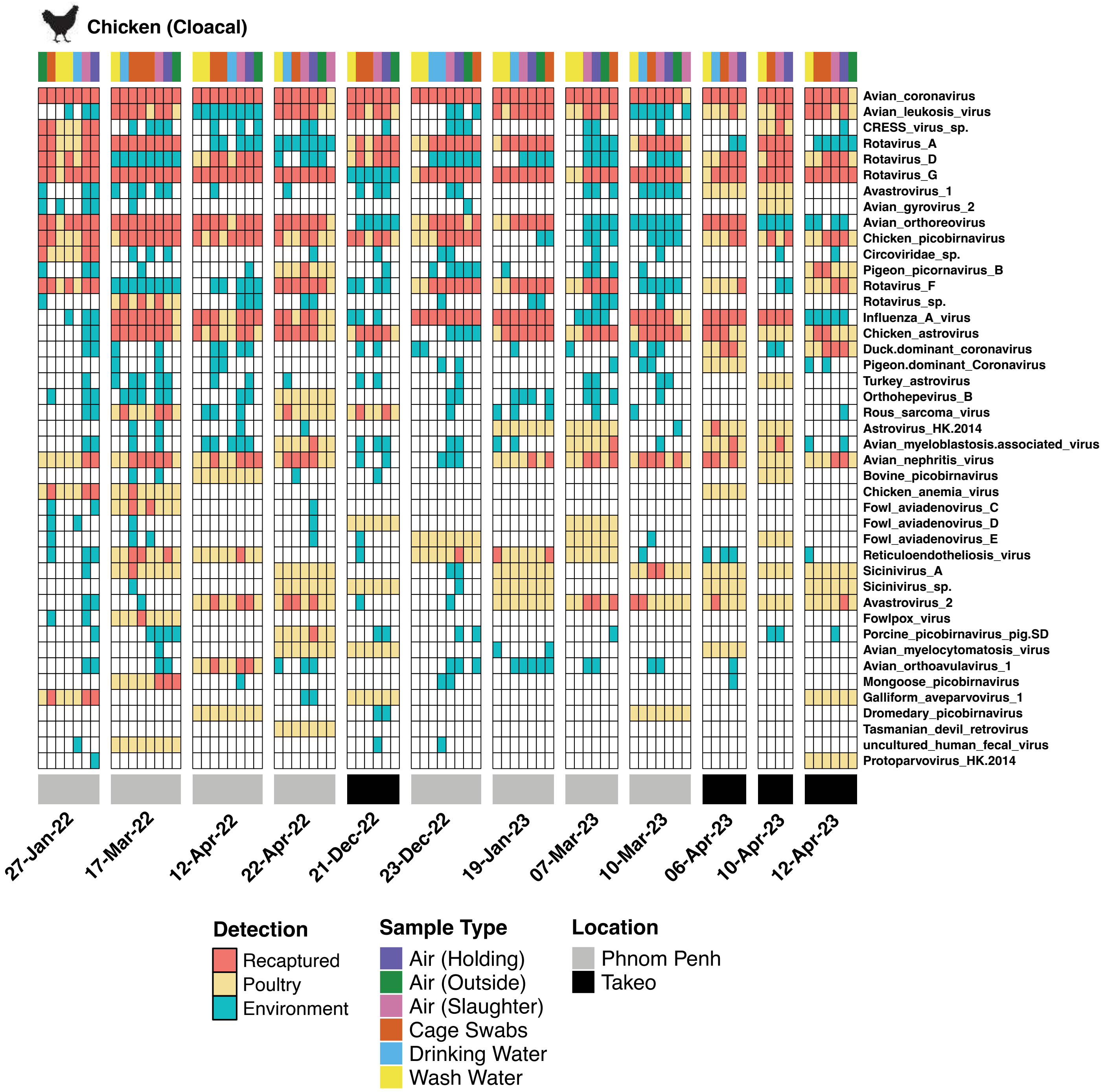

Supplementary Figure 6. Detection of chicken cloacal viruses through environmental sampling. Heatmap showing 43 different virus species which were detected at least once in chicken cloacal swabs over the course of the study. The red color shows when a virus was identified in chicken cloacal swabs and at the exact same timepoint was recaptured in the environment. The pale yellow colour shows when a virus species was identified in chicken cloacal swabs but failed to be recaptured through environmental sampling. The blue colour shows virus species which were found in the environment but failed to be detected in chicken cloacal swabs. Rows of the heatmap represent virus species and columns represent individual environmental samples (annotated at the top of the heatmap). The location of each timepoint is annotated on the bottom of the heatmap.

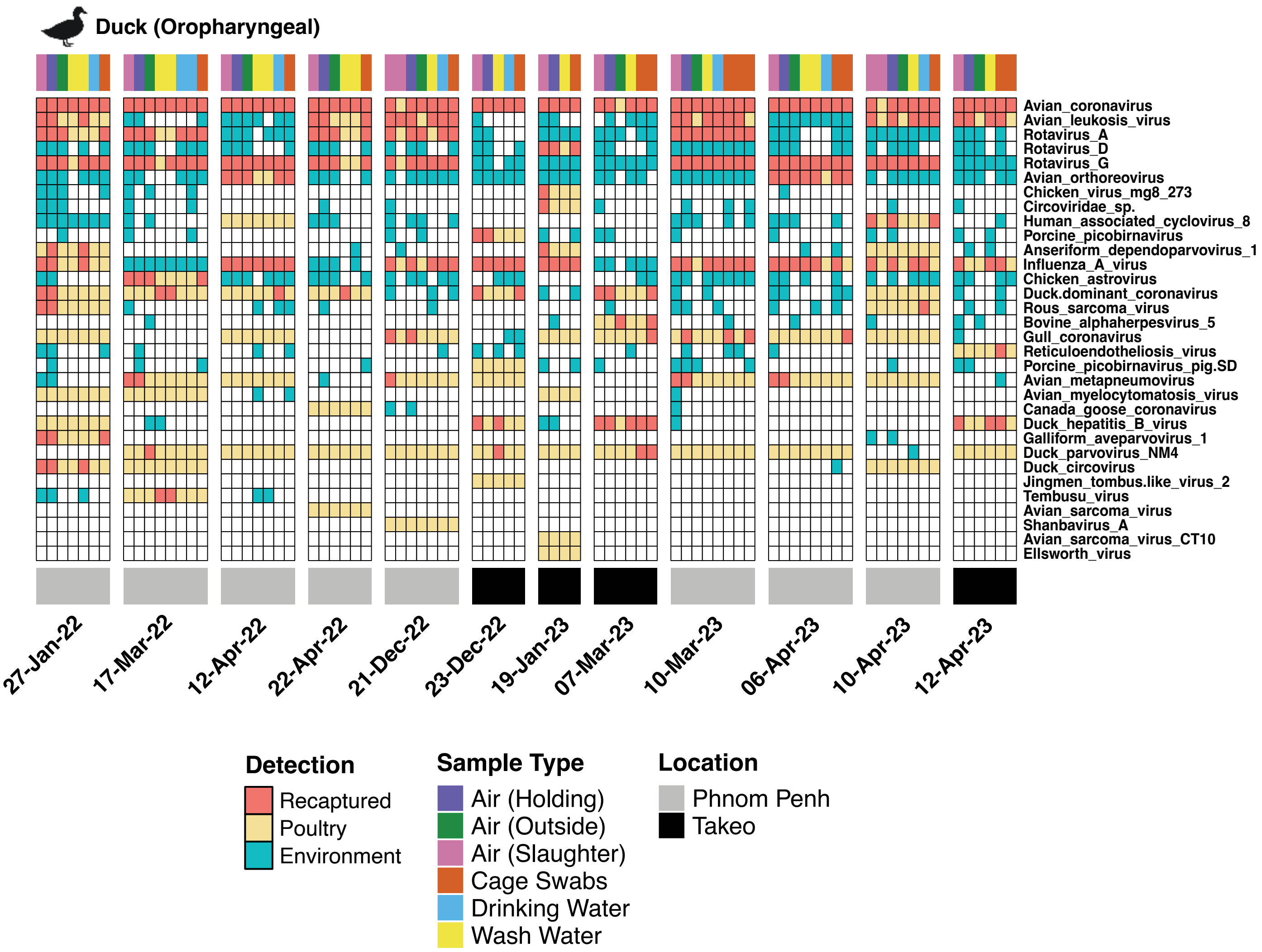

Supplementary Figure 7. Detection of duck oropharyngeal viruses through environmental sampling. Heatmap showing 32 different virus species which were detected at least once in duck oropharyngeal swabs over the course of the study. The red color shows when a virus was identified in duck oropharyngeal swabs and at the exact same timepoint was recaptured in the environment. The pale yellow colour shows when a virus species was identified in duck oropharyngeal swabs but failed to be recaptured through environmental sampling. The blue colour shows virus species which were found in the environment but failed to be detected in duck oropharyngeal swabs. Rows of the heatmap represent virus species and columns represent individual environmental samples (annotated at the top of the heatmap). The location of each timepoint is annotated on the bottom of the heatmap.

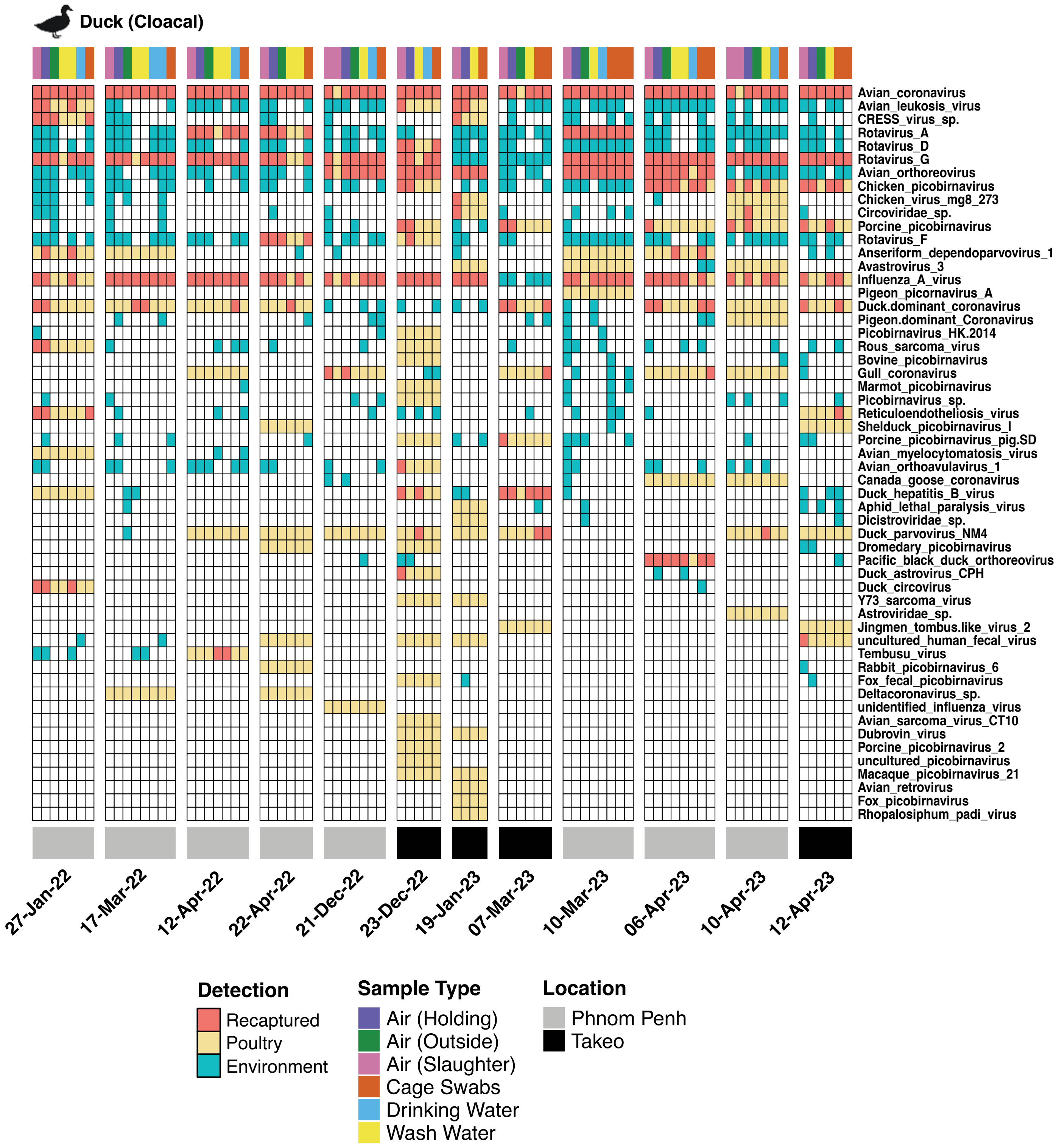

Supplementary Figure 8. Detection of duck cloacal viruses through environmental sampling. Heatmap showing 43 different virus species which were detected at least once in duck cloacal swabs over the course of the study. The red color shows when a virus was identified in duck cloacal swabs and at the exact same timepoint was recaptured in the environment. The pale yellow colour shows when a virus species was identified in duck cloacal swabs but failed to be recaptured through environmental sampling. The blue colour shows virus species which were found in the environment but failed to be detected in duck cloacal swabs. Rows of the heatmap represent virus species and columns represent individual environmental samples (annotated at the top of the heatmap). The location of each timepoint is annotated on the bottom of the heatmap.

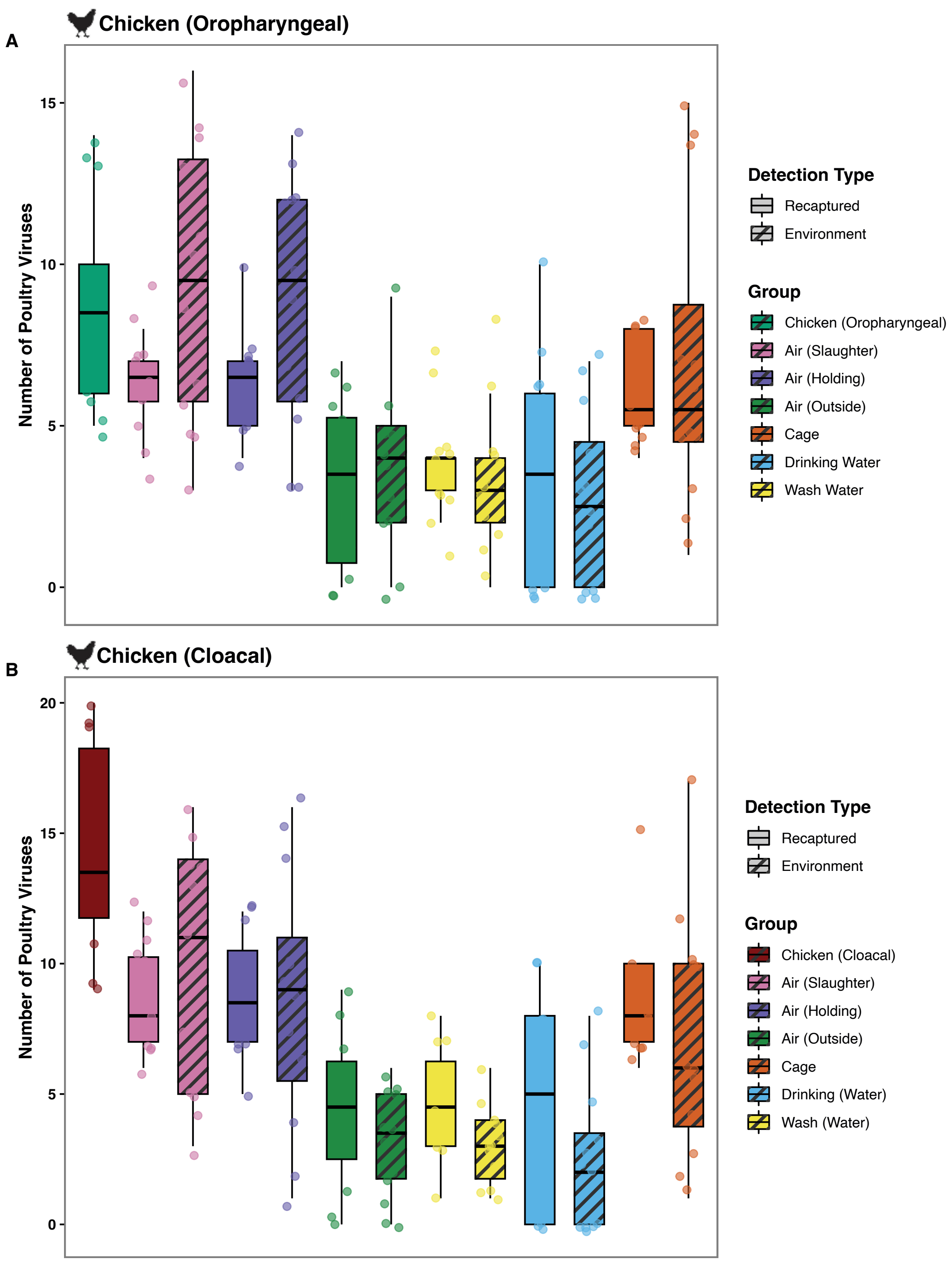

Supplementary Figure 9. Environmental samples recapture poultry viruses found in chicken swabs. The number of poultry viruses detected in A) chicken oropharyngeal and B) chicken cloacal swabs is compared to the number of the same poultry viruses recaptured in the environment and the number of additional poultry viruses found in the environment but not in poultry swabs. Statistics were calculated using a Kruskal–Wallis with Dunn's post-hoc test. All P-values obtained were corrected for false discovery rate (FDR) using the Benjamini–Hochberg method. P-values for all pairwise comparisons can be found in Supplementary Tables 6–7.

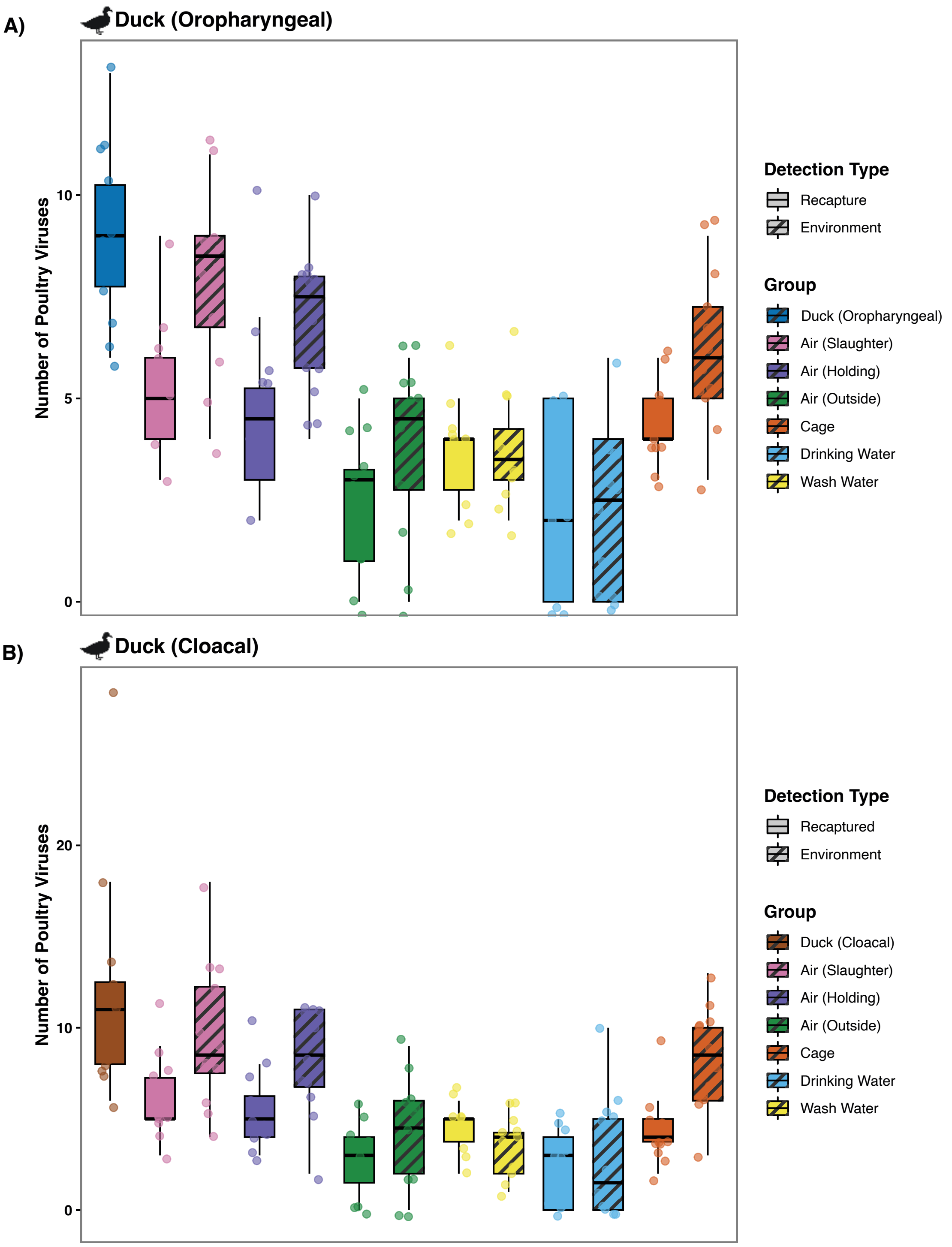

Supplementary Figure 10. Environmental samples recapture poultry viruses found in duck swabs. The number of poultry viruses detected in A) duck oropharyngeal and B) duck cloacal swabs is compared to the number of the same poultry viruses recaptured in the environment and the number of additional poultry viruses found in the environment but not in duck swabs. The latter is shown as a patterned boxplot. Statistics were calculated using a Kruskal–Wallis with Dunn's post-hoc test. All P-values obtained were corrected for false discovery rate (FDR) using the Benjamini–Hochberg method. P-values for all pairwise comparisons can be found in Supplementary Data 8–9.

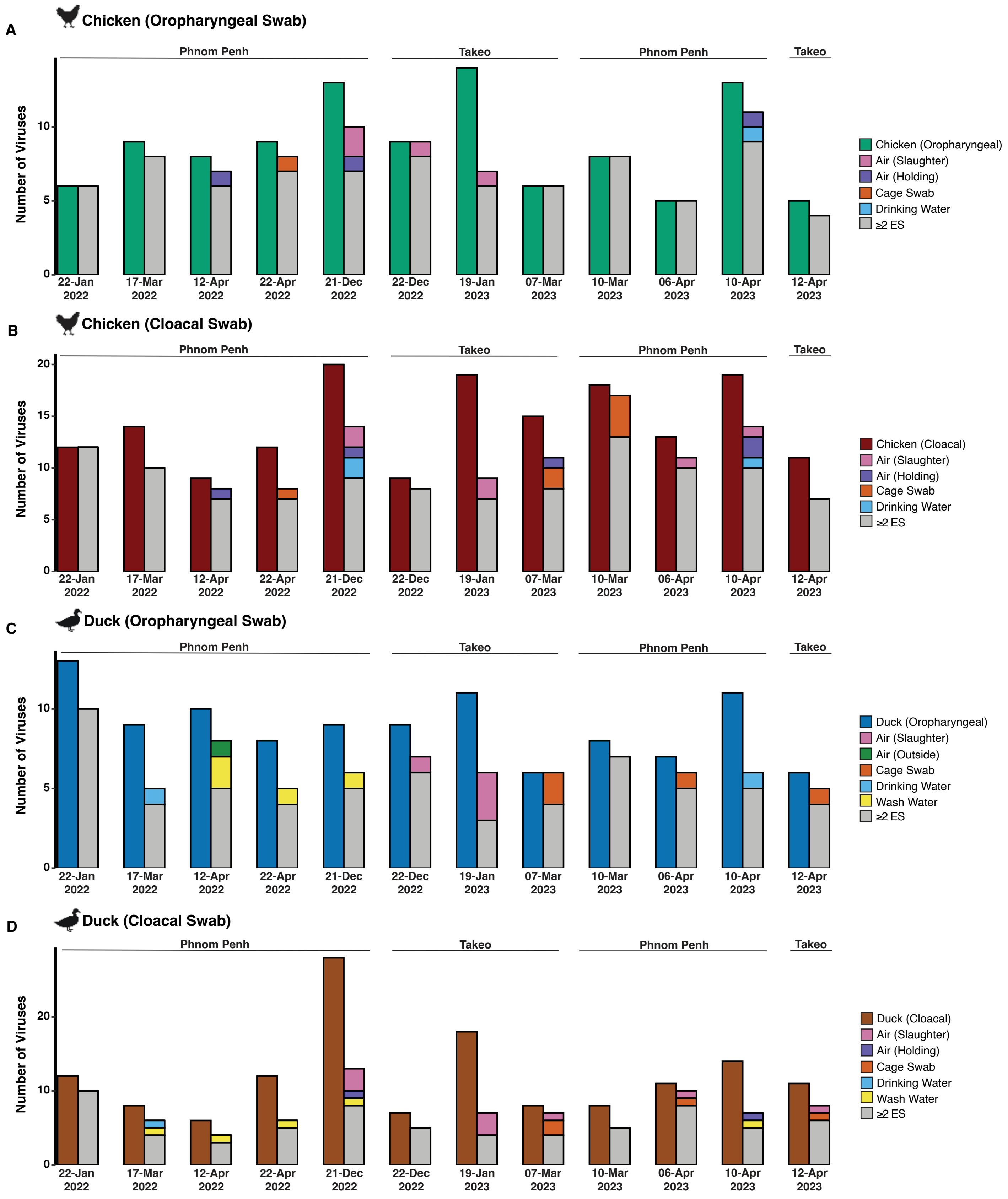

Supplementary Figure 11. A variety of environmental sample types are required to recapture the most poultry viruses. Stacked bar plots showing the number of viruses detected in A) chicken oropharyngeal, B) chicken cloacal, C) duck oropharyngeal and D) duck cloacal swabs compared to the environmental samples at each timepoint. If a virus was recaptured in two or more environmental sample types, the bar is coloured grey. Poultry viruses uniquely recaptured in only one environmental sample type are colour-coded according to the legend on the right.

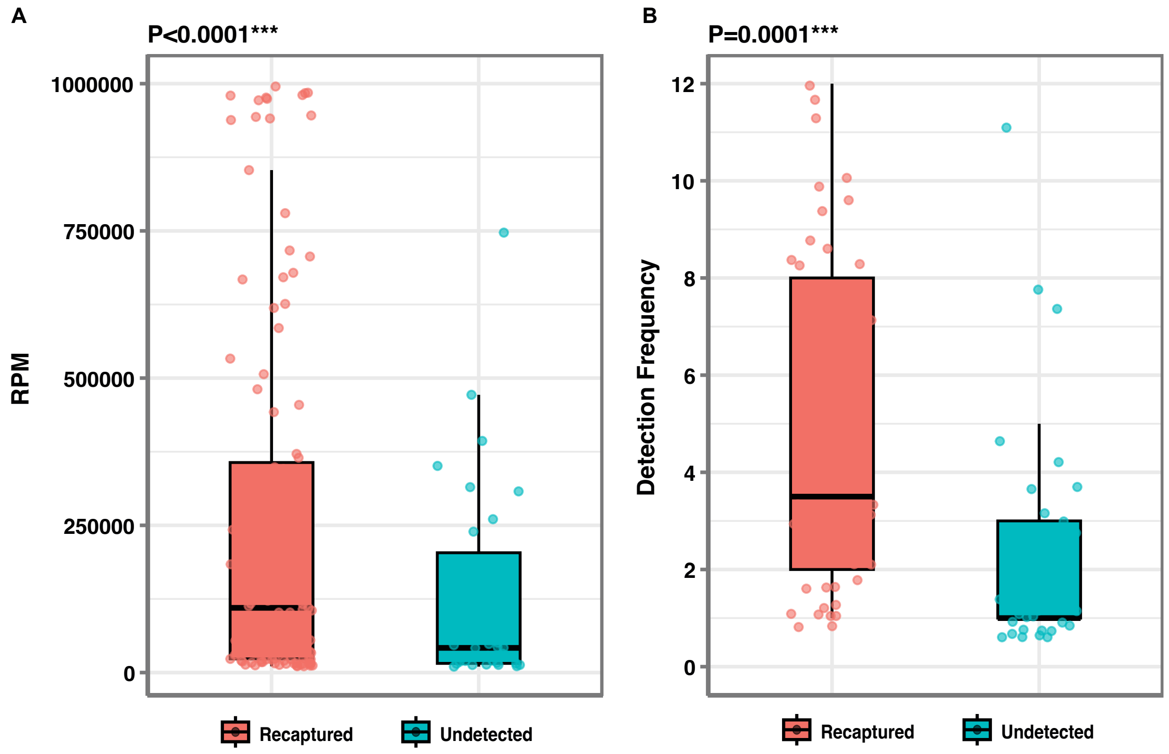

Supplementary Figure 12. Poultry viruses accurately recaptured in the environment had a higher abundance and appeared more frequently at LBMs in Cambodia.. Box plots comparing A) abundance measured in reads per million (RPM) and B) frequency of detection at LBMs comparing poultry viruses which were successfully recaptured through environmental sampling and viruses which went undetected. Statistical significance was calculated using Wilcoxon test. All P-values obtained were corrected for false discovery rate (FDR) using the Benjamini–Hochberg method. P-values are annotated as follows: P < 0.05 *; P < 0.01 **; P < 0.001 ***.

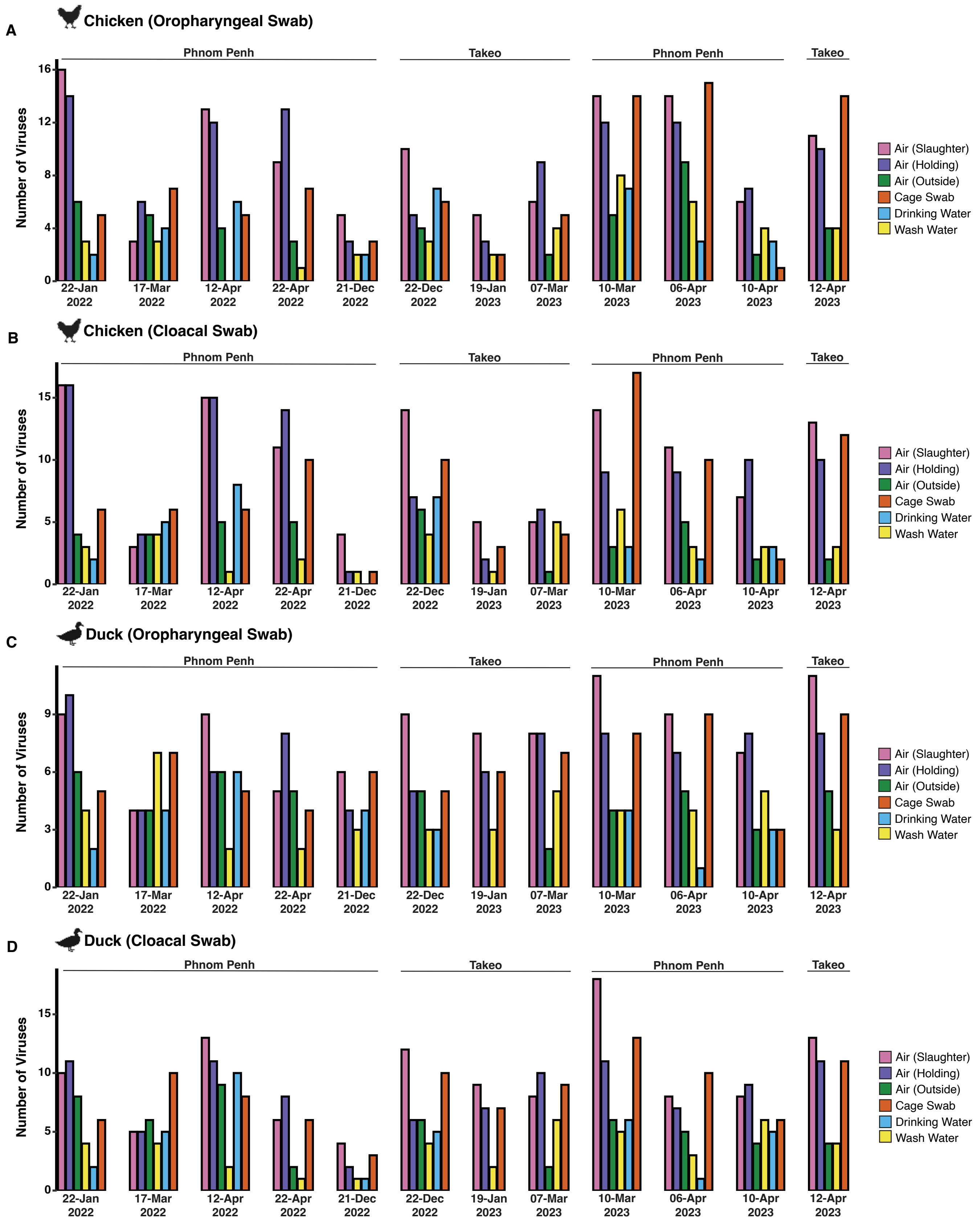

Supplementary Figure 13. Environmental sampling detects a large number of domestic poultry viruses which failed to be identified through poultry swab sampling. Bar plots showing the number of additional A) chicken oropharyngeal, B) chicken cloacal, C) duck oropharyngeal, and D) duck cloacal viruses detected exclusively in environmental samples at each timepoint. These data exclude recaptured viruses and represent only those detected in the environment but absent from poultry swabs at the same timepoint.

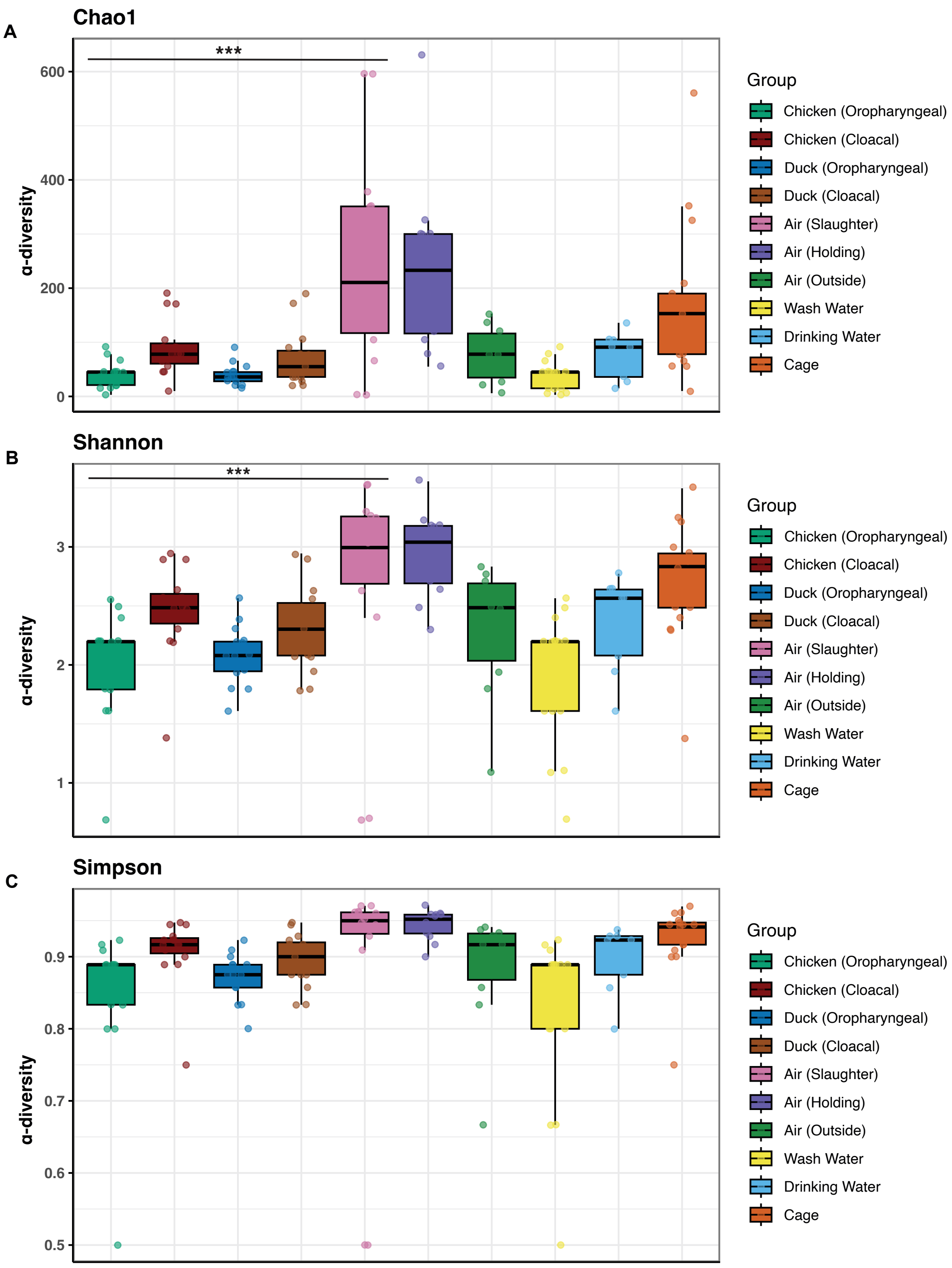

Supplementary Figure 14. Air samples have a higher alpha-diversity than poultry swabs. Box plots showing alpha-diversity measures A) Chao1, B) Shannon, and C) Simpson compared between each group. Samples from all timepoints are included. Statistics were calculated using a Kruskal–Wallis with Dunn's post-hoc test. All P-values obtained were corrected for false discovery rate (FDR) using the Benjamini–Hochberg method. P-values for all pairwise comparisons can be found in Supplementary Table 11–13. P-values are annotated as follows: P < 0.05 *; P < 0.01 **; P < 0.001 ***.

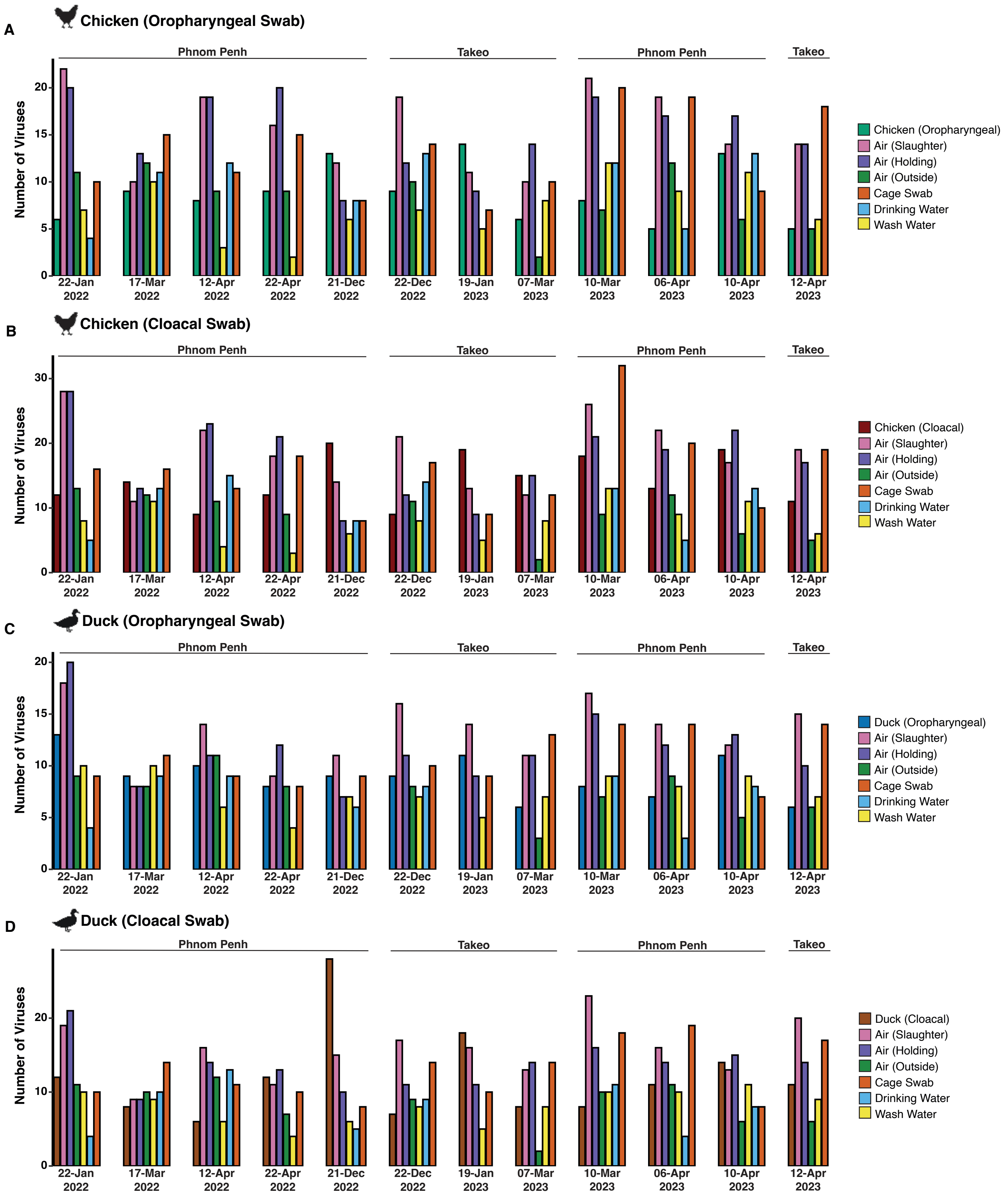

Supplementary Figure 15. Enhanced detection of poultry viruses through environmental metagenomics.. Barplots showing the total number of viruses detected in A) chicken oropharyngeal, B) chicken cloacal, C) duck oropharyngeal, and D) duck cloacal swabs compared to environmental samples at each timepoint. For each environmental sample type, the totals include only those viruses that have been detected at least once in the corresponding poultry swab type to which it is compared. Thus, each total comprises viruses recaptured from the corresponding poultry swab and additional viruses detected only in the environment at that timepoint.

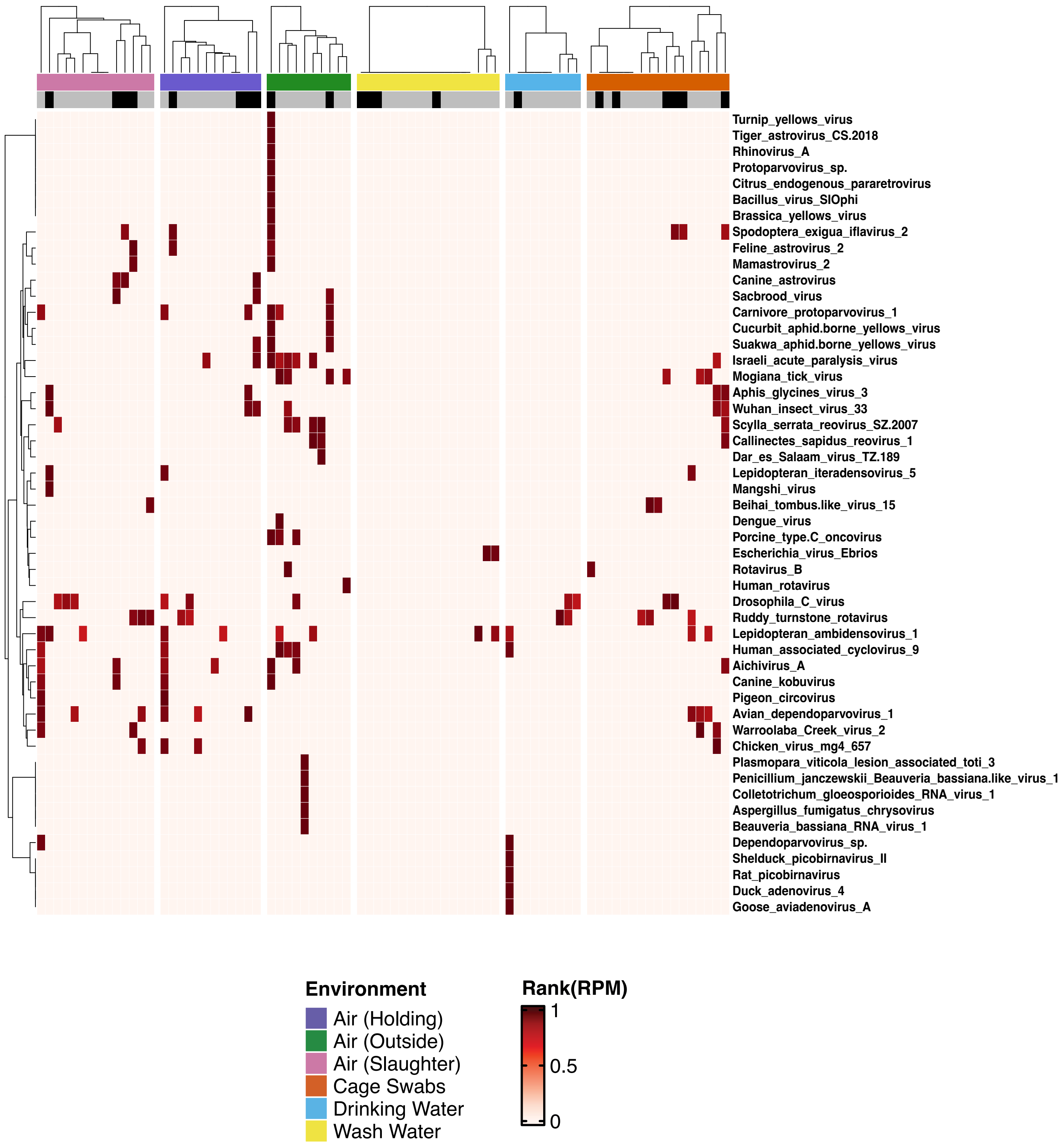

Supplementary Figure 16. Additional viruses identified through metagenomics of environmental samples which could not be found in poultry. Heatmap showing the abundance of non-poultry viruses detected, represented as ranked reads per million (RPMs). Rows represent different viruses, while columns indicate sampling locations and environmental sample types.

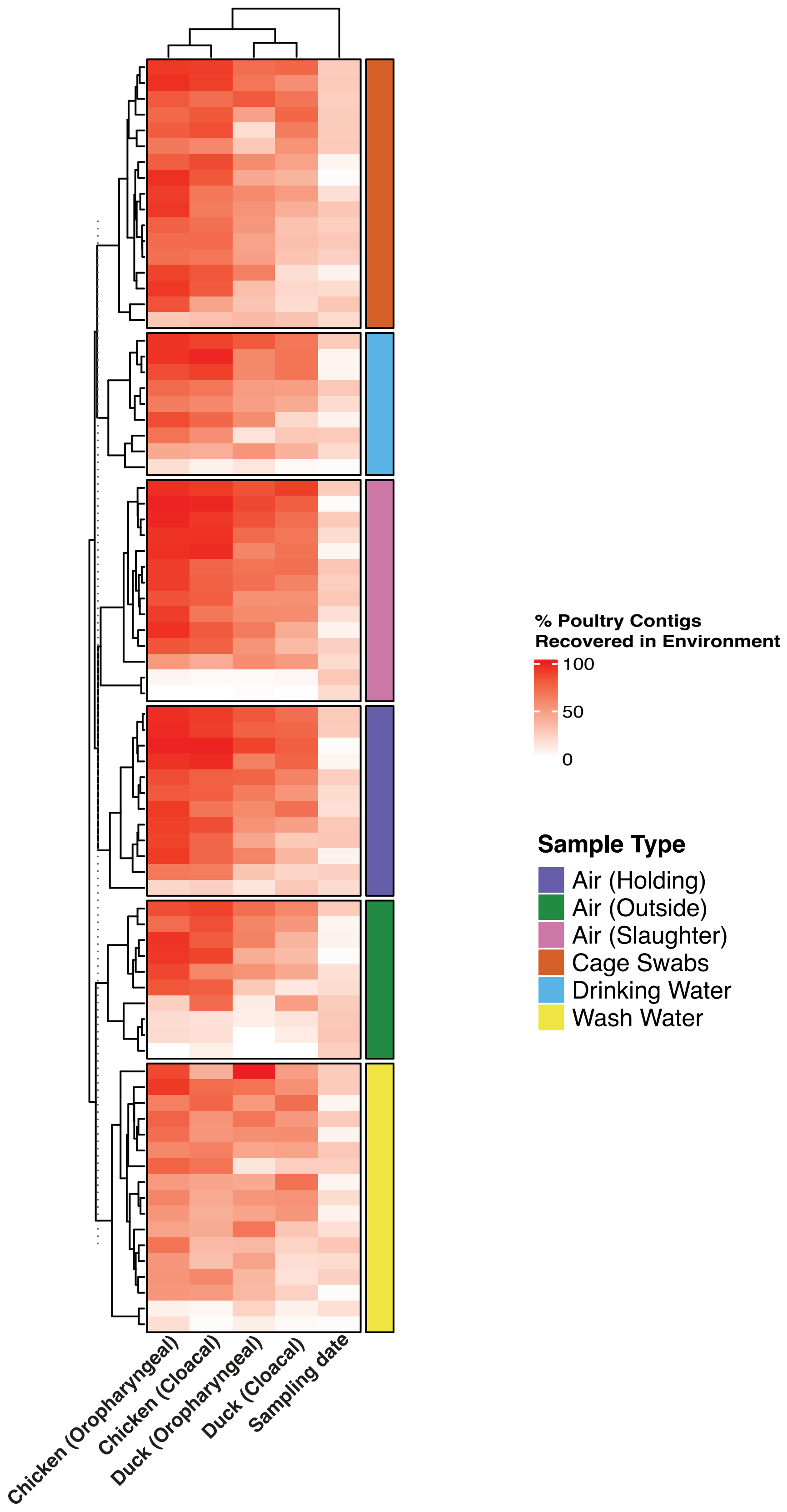

Supplementary Figure 17. The majority of poultry viral contigs aligned to those assembled from environmental samples. The heatmap displays the percentage of poultry contigs mapping to environmental contigs. Each row represents an environmental sample (annotated by colour on the right), while columns correspond to poultry samples and their respective sampling dates.

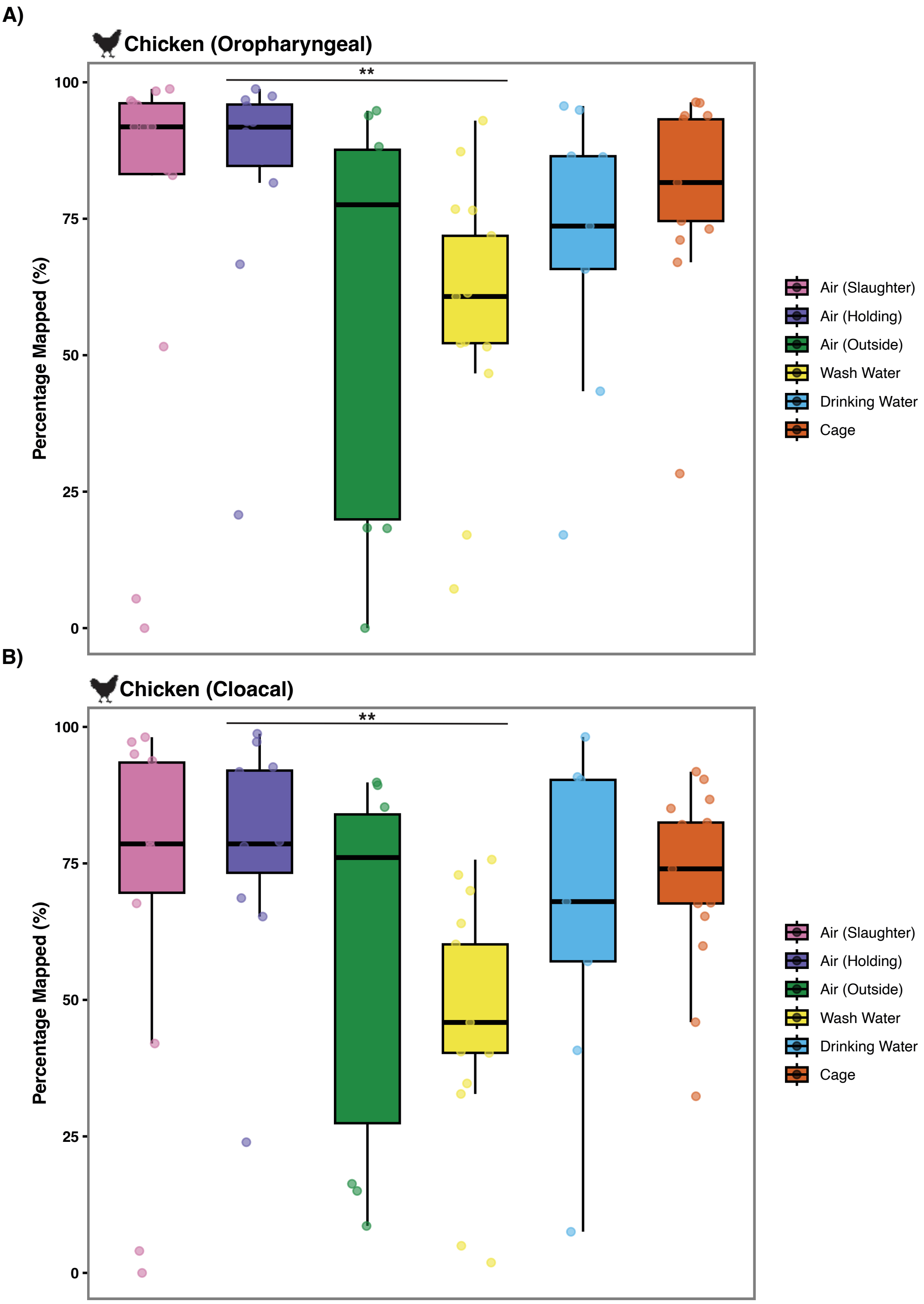

Supplementary Figure 18. The majority of contigs assembled from chicken samples align to contigs from the environment. Alignment rates for A) oropharyngeal and B) cloacal swabs collected from chickens are shown for each individual environmental sample. Statistics were calculated using a Kruskal–Wallis with Dunn's post-hoc test. All P-values obtained were corrected for false discovery rate (FDR) using the Benjamini–Hochberg method. P-values for all pairwise comparisons can be found in Supplementary Data 23–24. P-values are annotated as follows: $P < 0.05$ *; $P < 0.01$ **; $P < 0.001$ ***.

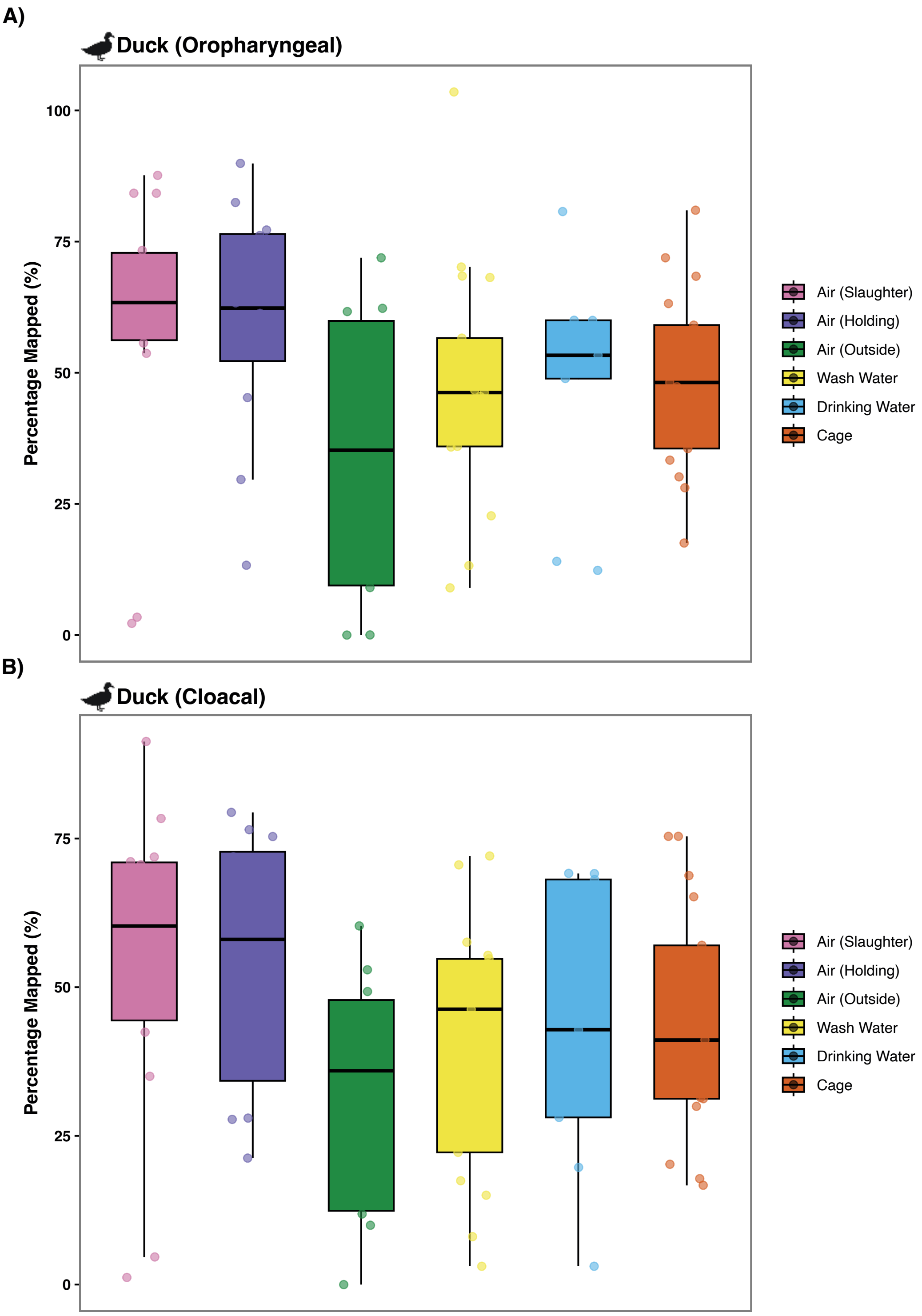

Supplementary Figure 19. The majority of contigs assembled from duck samples align to contigs from the environment. Alignment rates for A) oropharyngeal and B) cloacal swabs collected from ducks are shown for each individual environmental sample. Statistics were calculated using a Kruskal–Wallis with Dunn's post-hoc test. All P-values obtained were corrected for false discovery rate (FDR) using the Benjamini–Hochberg method. P-values for all pairwise comparisons can be found in Supplementary Data 25–26.

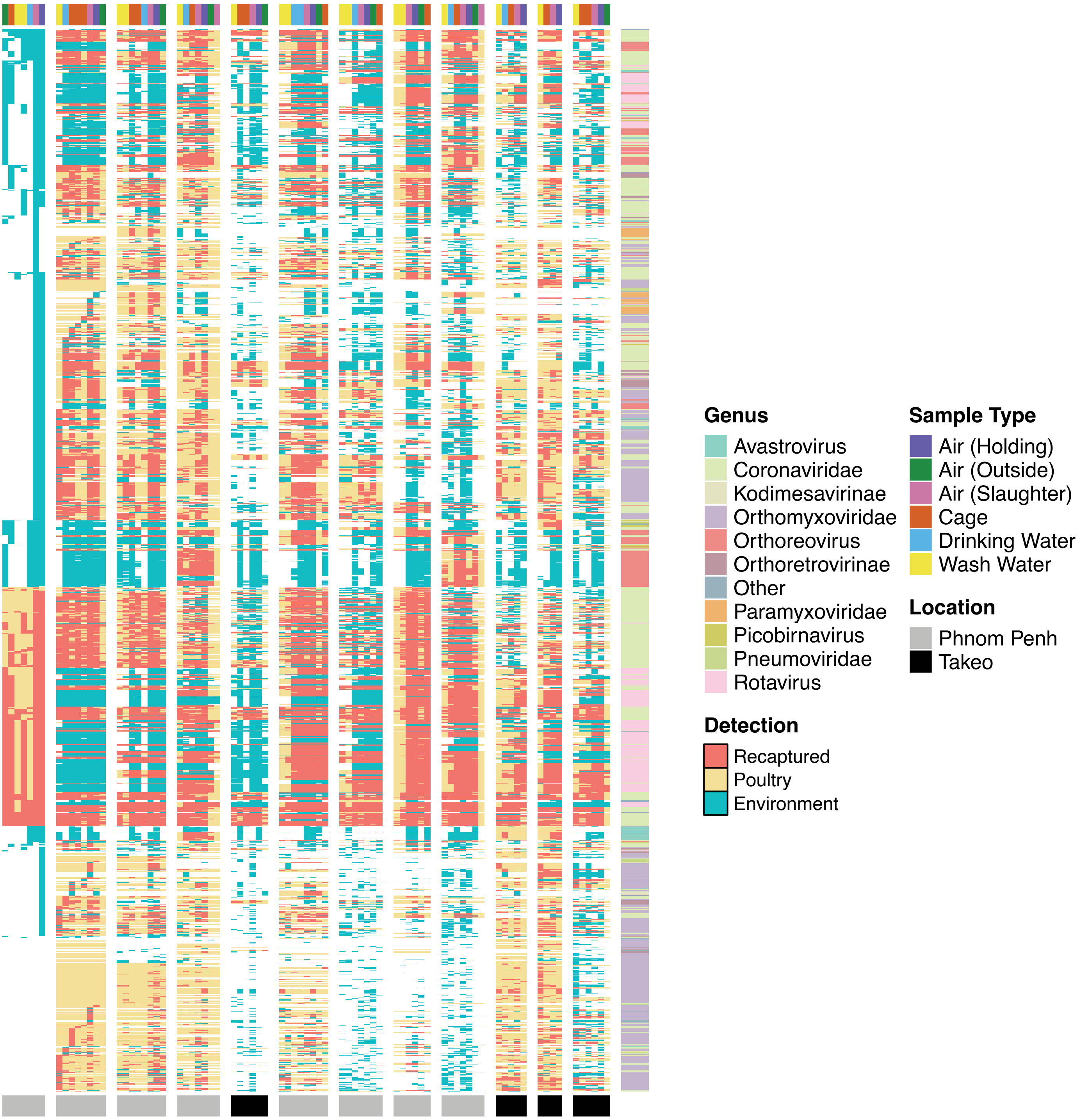

Supplementary Figure 20. Detection of virus genes associated with chicken oropharyngeal swabs through environmental sampling. Heatmap showing different virus genes which were detected at least once in chicken oropharyngeal swabs over the course of the study. The red color shows when a viral gene was identified in chicken oropharyngeal swabs and at the exact same timepoint was recaptured in the environment. The pale yellow colour shows when a virus gene was identified in chicken oropharyngeal swabs but failed to be recaptured through environmental sampling. The blue colour shows virus genes which were found in the environment but failed to be detected in chicken oropharyngeal swabs. Rows of the heatmap represent individual virus genes and columns represent individual environmental samples (annotated at the top of the heatmap). The location of each timepoint is annotated on the bottom of the heatmap.

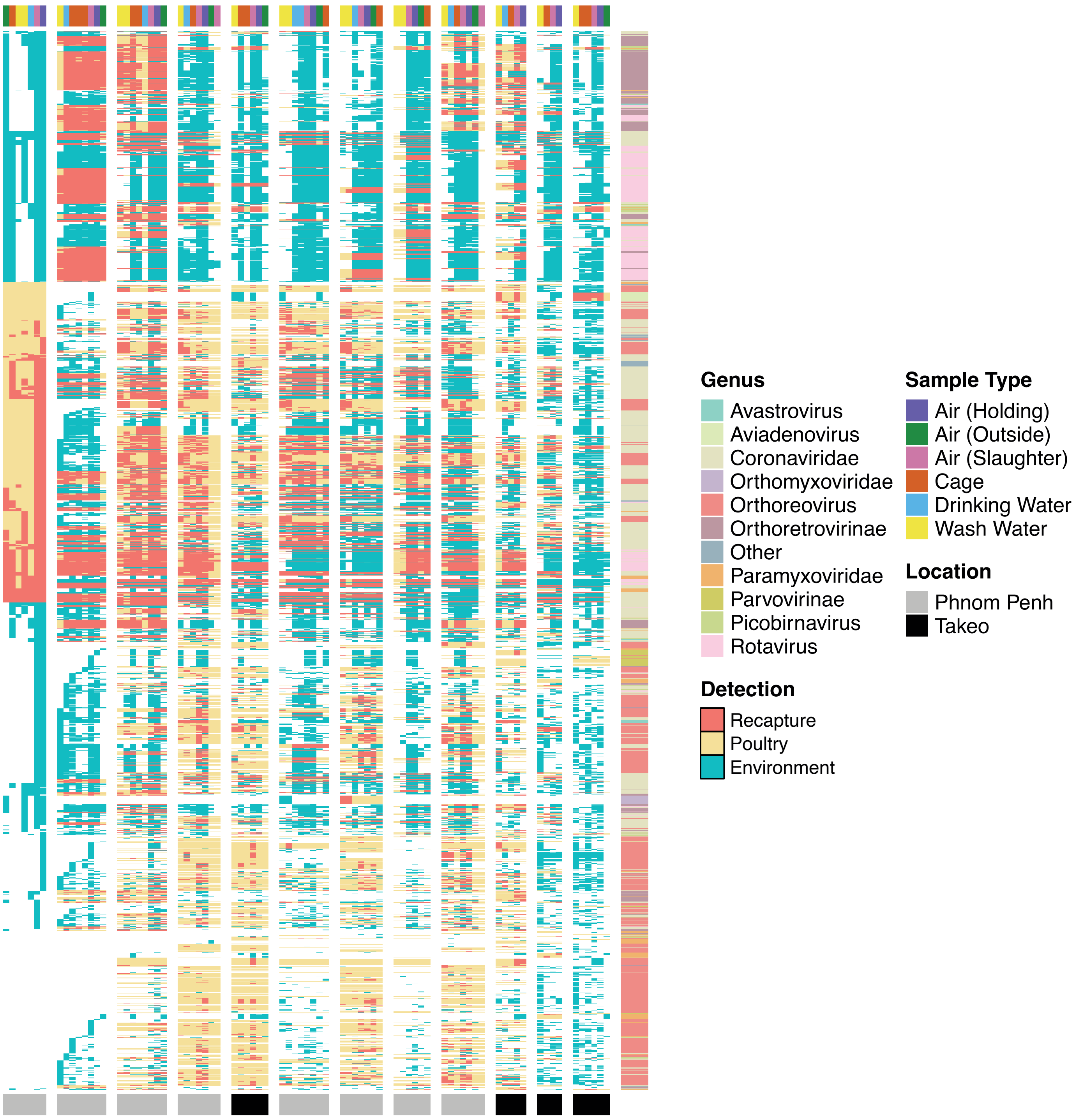

Supplementary Figure 21. Detection of virus genes associated with chicken cloacal swabs through environmental sampling. Heatmap showing different virus genes which were detected at least once in chicken cloacal swabs over the course of the study. The red color shows when a viral gene was identified in chicken cloacal swabs and at the exact same timepoint was recaptured in the environment. The pale yellow colour shows when a virus gene was identified in chicken cloacal swabs but failed to be recaptured through environmental sampling. The blue colour shows virus genes which were found in the environment but failed to be detected in chicken cloacal swabs. Rows of the heatmap represent individual virus genes and columns represent individual environmental samples (annotated at the top of the heatmap). The location of each timepoint is annotated on the bottom of the heatmap.

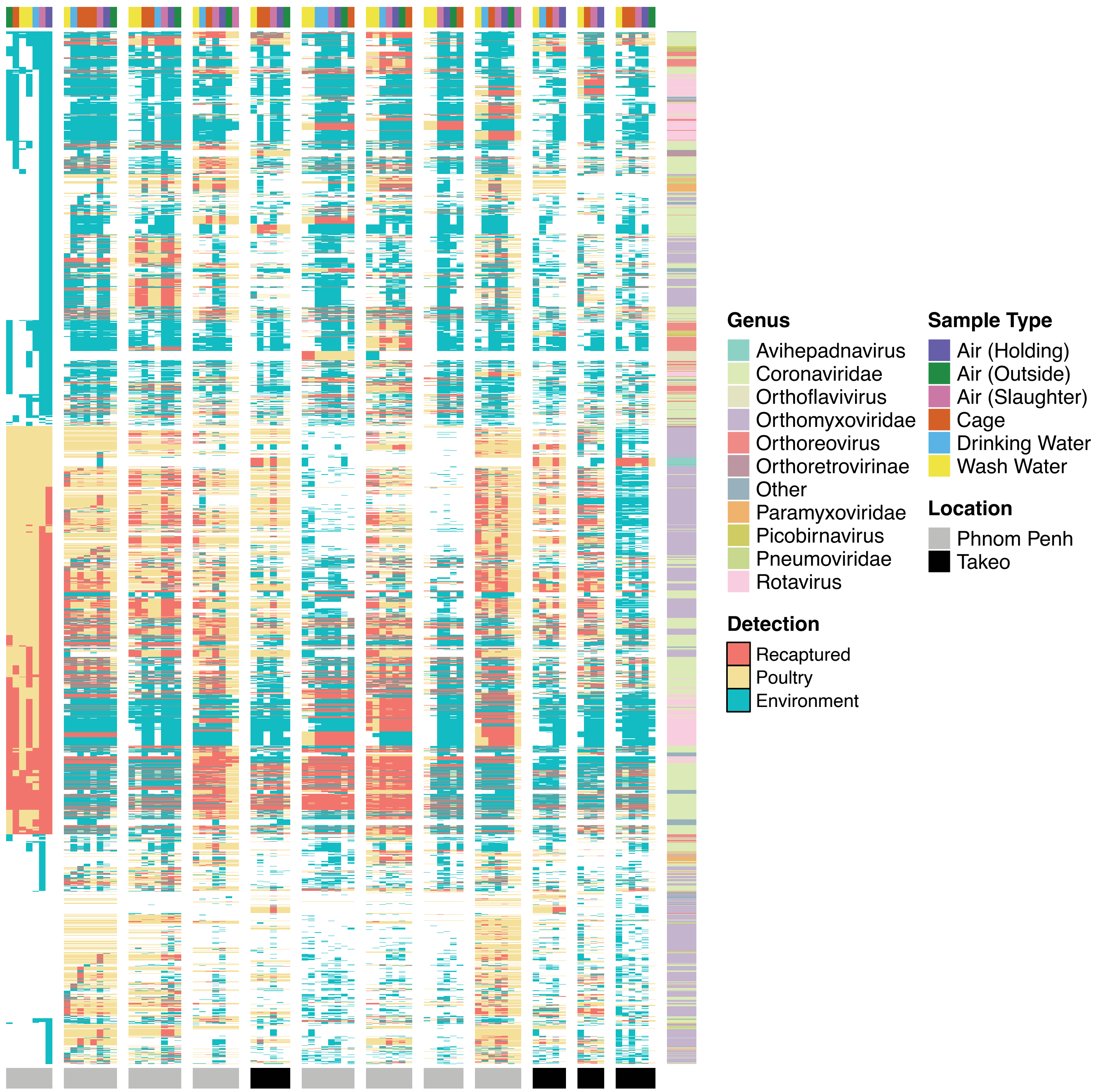

Supplementary Figure 22. Detection of virus genes associated with duck throat swabs through environmental sampling. Heatmap showing different virus genes which were detected at least once in duck throat swabs over the course of the study. The red color shows when a viral gene was identified in duck throat swabs and at the exact same timepoint was recaptured in the environment. The pale yellow colour shows when a virus gene was identified in duck throat swabs but failed to be recaptured through environmental sampling. The blue colour shows virus genes which were found in the environment but failed to be detected in duck throat swabs. Rows of the heatmap represent individual virus genes and columns represent individual environmental samples (annotated at the top of the heatmap). The location of each timepoint is annotated on the bottom of the heatmap.

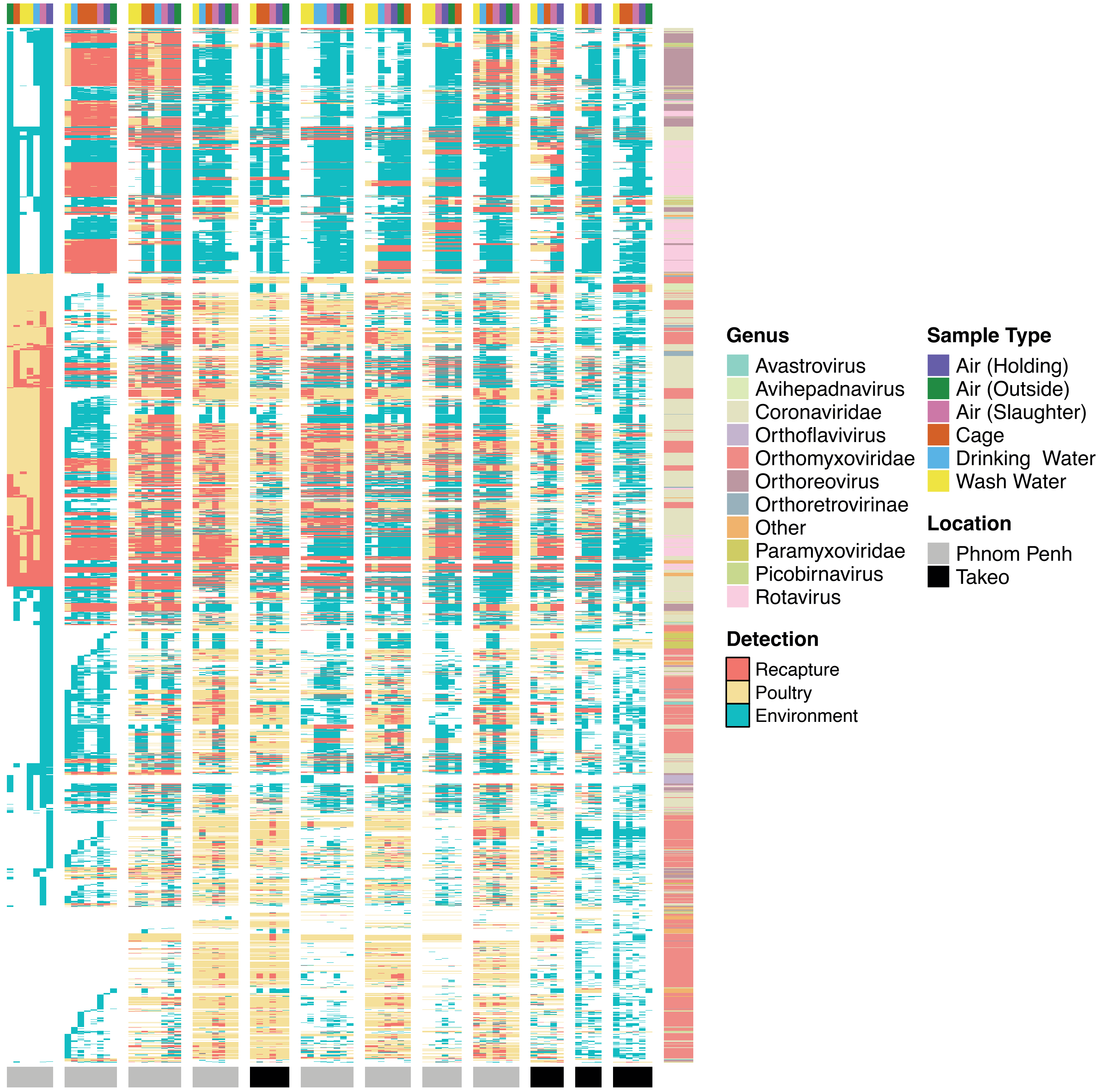

Supplementary Figure 23. Detection of virus genes associated with duck cloacal swabs through environmental sampling. Heatmap showing different virus genes which were detected at least once in duck cloacal swabs over the course of the study. The red color shows when a viral gene was identified in duck cloacal swabs and at the exact same timepoint was recaptured in the environment. The pale yellow colour shows when a virus gene was identified in duck cloacal swabs but failed to be recaptured through environmental sampling. The blue colour shows virus genes which were found in the environment but failed to be detected in duck cloacal swabs. Rows of the heatmap represent individual virus genes and columns represent individual environmental samples (annotated at the top of the heatmap). The location of each timepoint is annotated on the bottom of the heatmap.

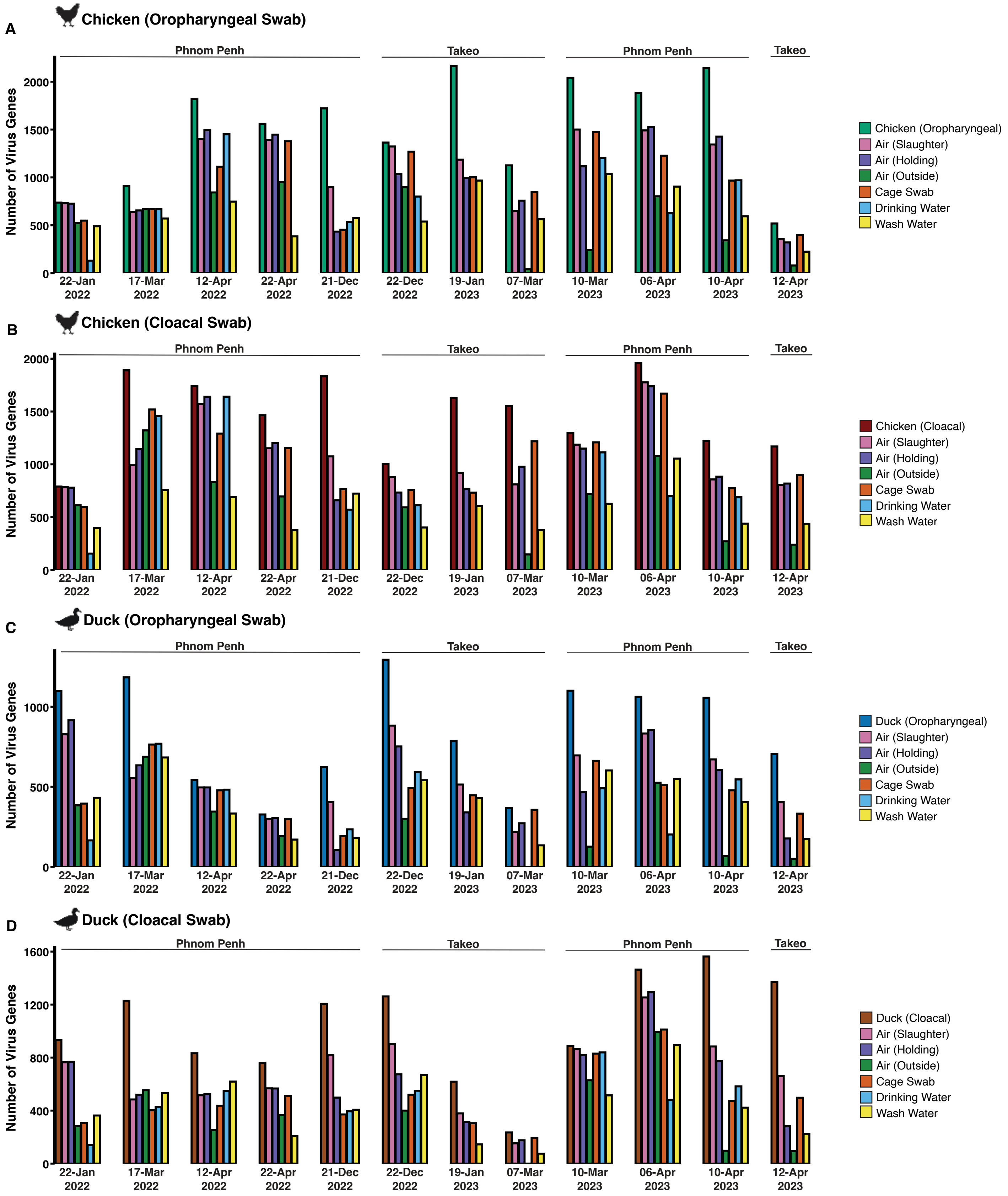

Supplementary Figure 24. Individual environmental samples recapture the majority of viral genes detected in different poultry swab types. Barplots showing the number of viral genes detected in A) chicken oropharyngeal, B) chicken cloacal, C) duck oropharyngeal, and D) duck cloacal swabs compared to the environmental samples at each timepoint. For each environmental sample type, the totals only include viral genes that were identified in the respective poultry swab type at that specific timepoint. Thus, the data shown focus solely on the ability of environmental samples to recapture what was found in poultry swabs and do not include additional poultry viruses detected at that time.

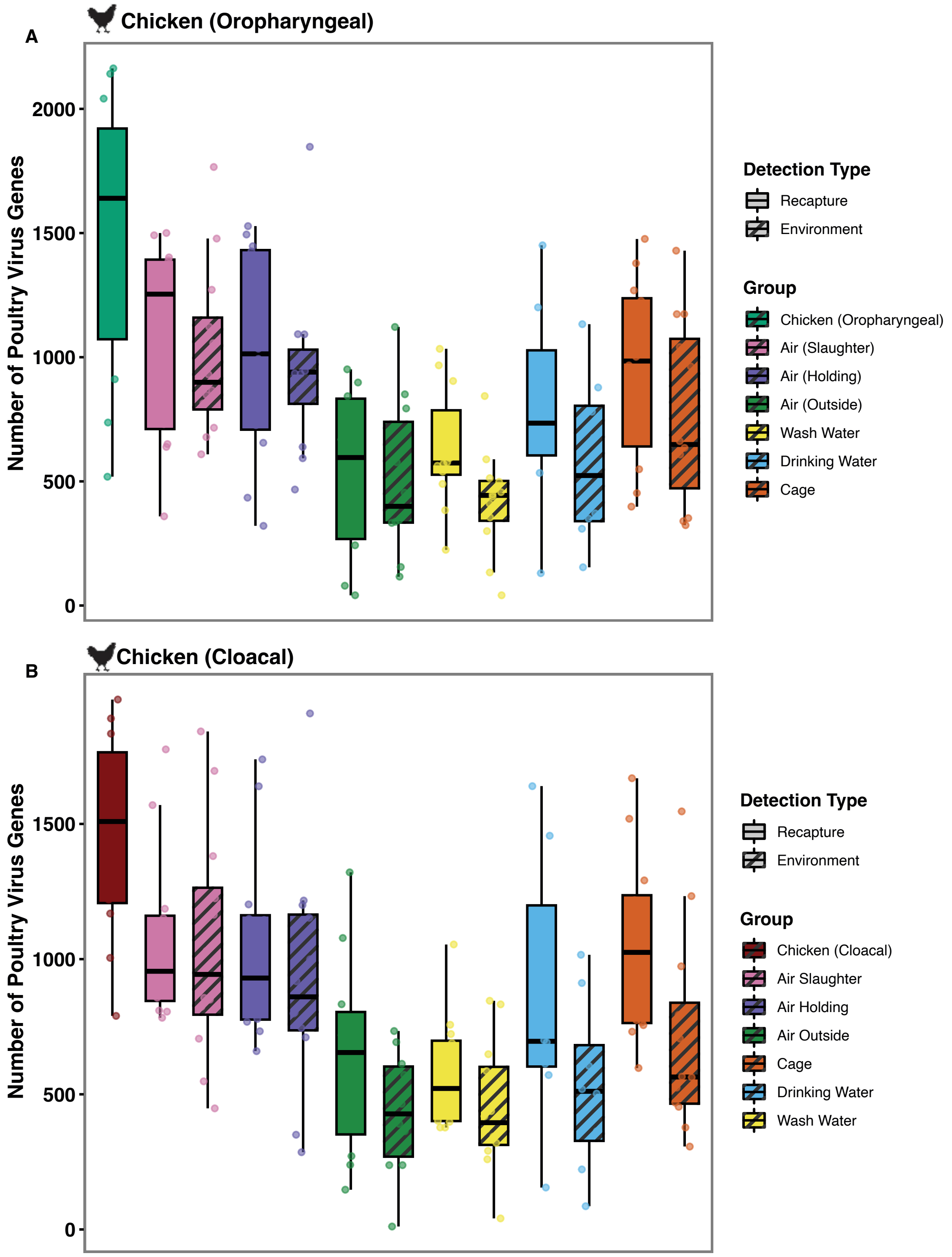

Supplementary Figure 25. Environmental samples recapture poultry virus genes found in chicken swabs. The number of poultry virus genes detected in A) chicken oropharyngeal and B) chicken cloacal swabs is compared to the number of the same poultry virus genes recaptured in the environment and the number of additional poultry virus genes found in the environment but not in poultry swabs. Statistics were calculated using a Kruskal–Wallis with Dunn's post-hoc test. All P-values obtained were corrected for false discovery rate (FDR) using the Benjamini–Hochberg method. P-values for all pairwise comparisons can be found in Supplementary Tables 27–28.

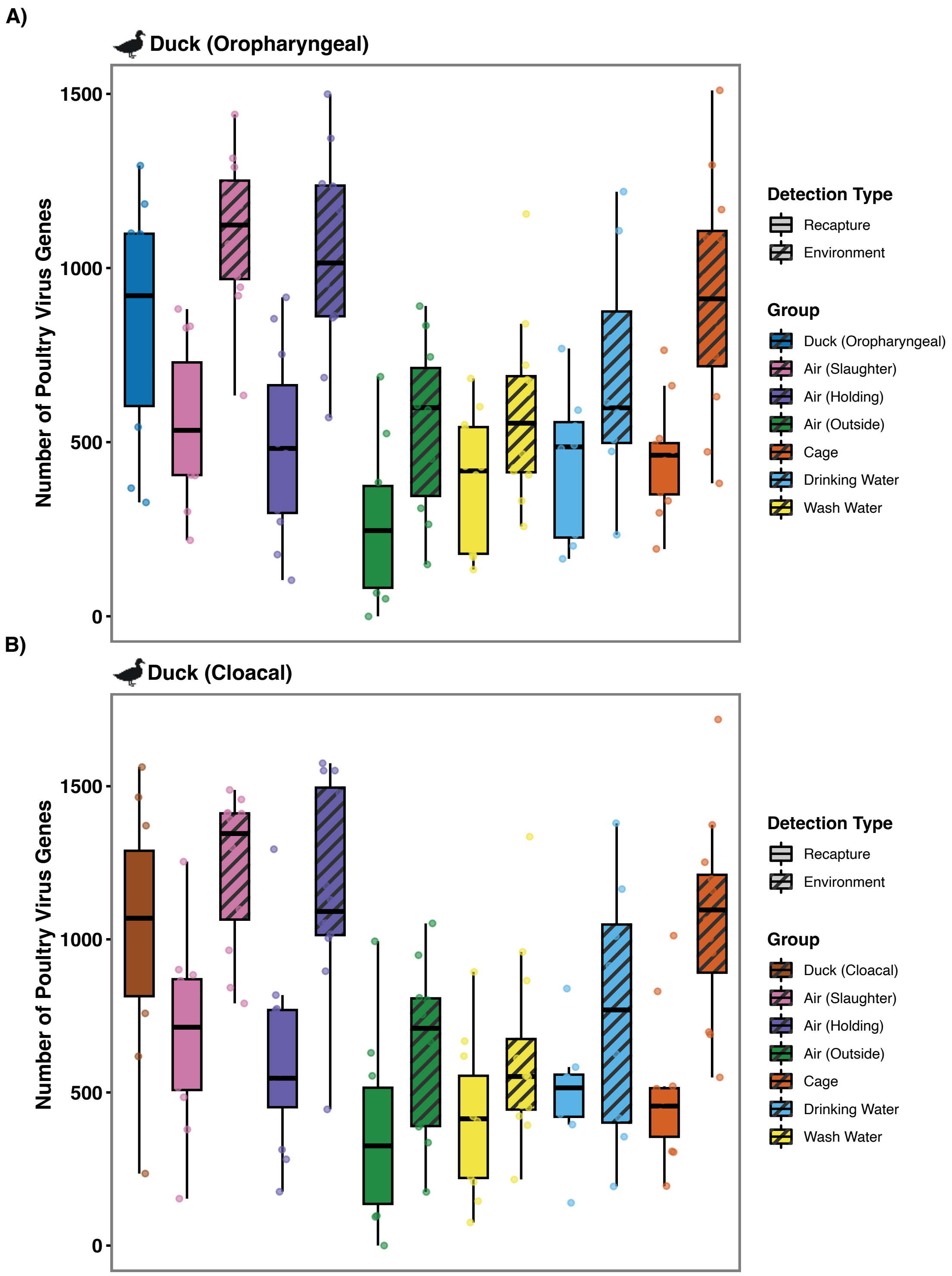

Supplementary Figure 26. Environmental samples recapture poultry virus genes found in duck swabs. The number of poultry virus genes detected in A) duck oropharyngeal and B) duck cloacal swabs is compared to the number of the same poultry virus genes recaptured in the environment and the number of additional poultry virus genes found in the environment but not in duck swabs. The latter is shown as a patterned boxplot. Statistics were calculated using a Kruskal–Wallis with Dunn's post-hoc test. All P-values obtained were corrected for false discovery rate (FDR) using the Benjamini–Hochberg method. P-values for all pairwise comparisons can be found in Supplementary Data 29–30.

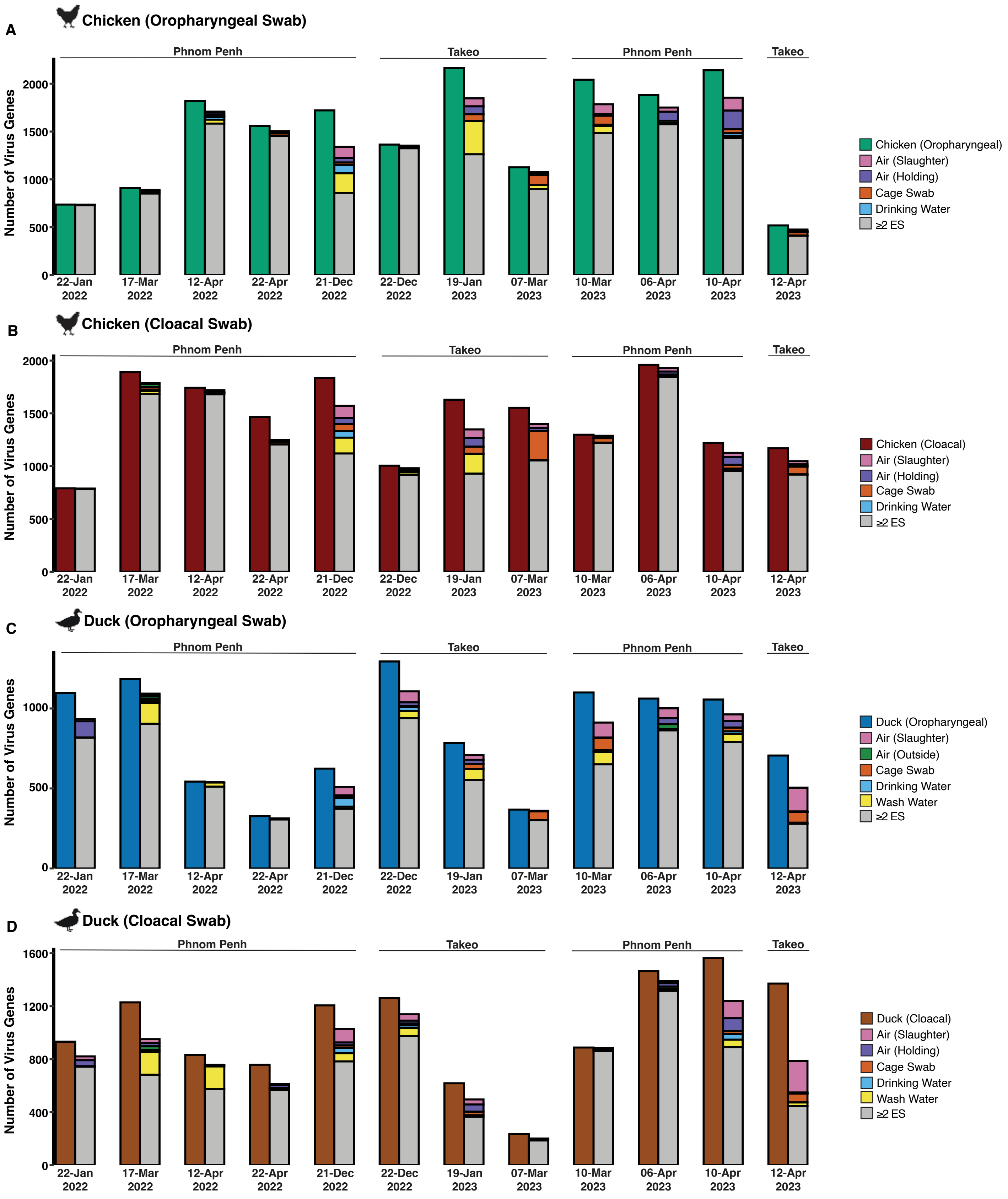

Supplementary Figure 27. A variety of environmental sample types are required to recapture the most poultry virus genes. Stacked bar plots showing the number of virus genes detected in A) chicken oropharyngeal, B) chicken cloacal, C) duck oropharyngeal, and D) duck cloacal swabs compared to the environmental samples at each timepoint. If a virus gene was recaptured in two or more environmental sample types, the bar is coloured grey. Poultry virus genes uniquely recaptured in only one environmental sample type are colour-coded according to the legend on the right.

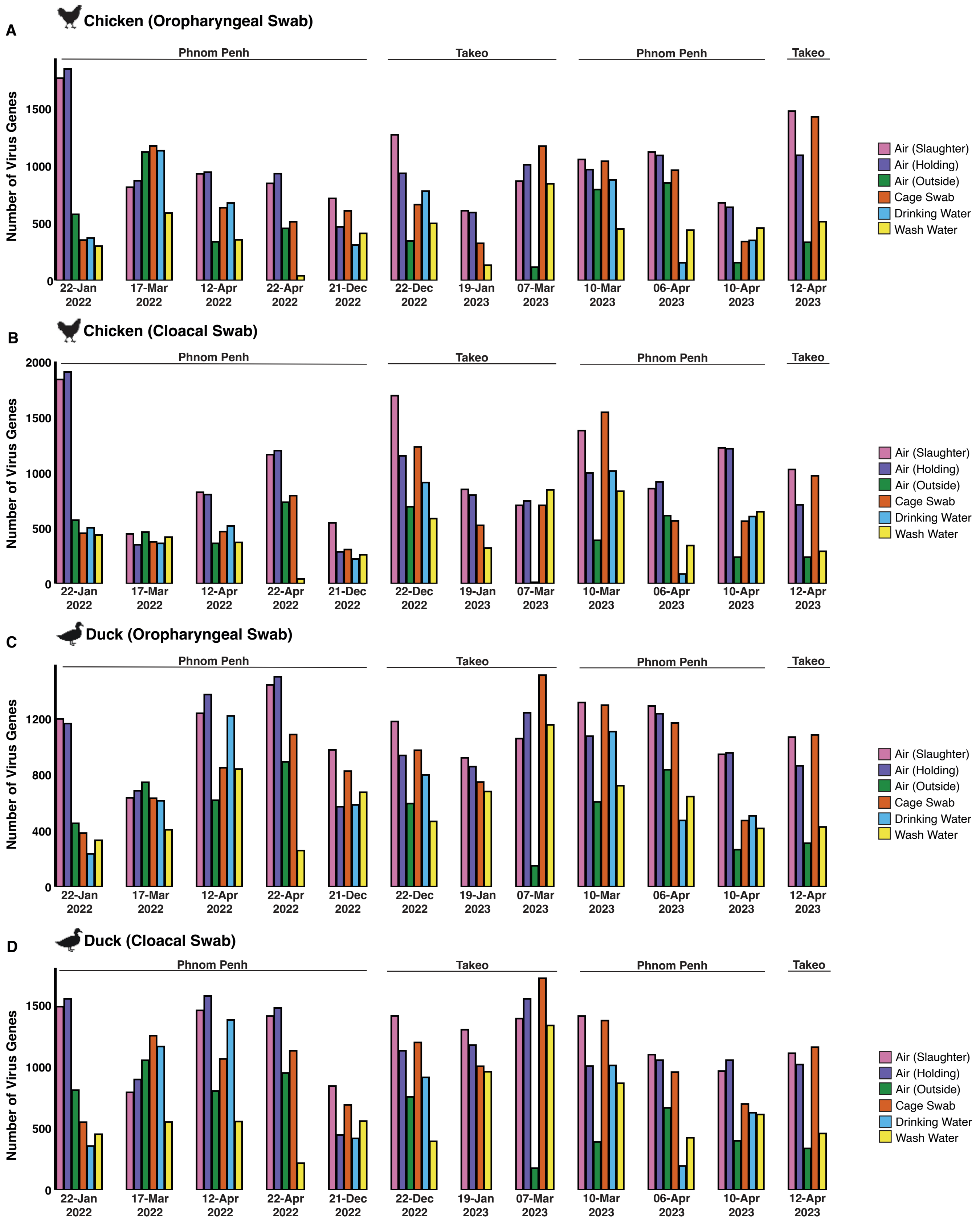

Supplementary Figure 28. Contigs from environmental sampling represent a large number of domestic poultry virus genes which failed to be detected in poultry samples. Bar plots showing the number of additional A) chicken oropharyngeal, B) chicken cloacal, C) duck oropharyngeal, and D) duck cloacal virus genes detected exclusively in environmental samples at each timepoint. These data exclude recaptured virus genes and represent only those detected in the environment but absent from poultry swabs at the same timepoint.

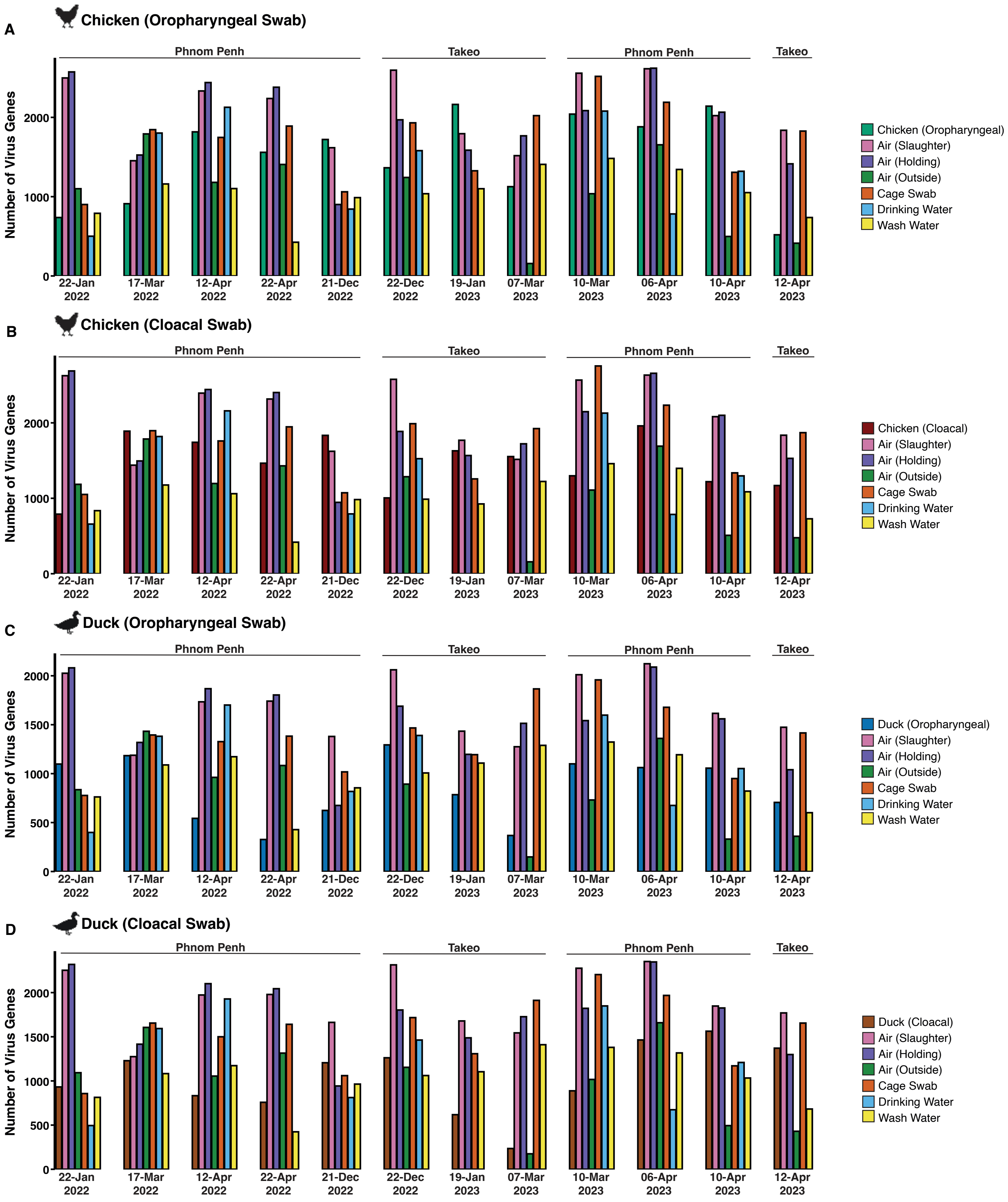

Supplementary Figure 29. Enhanced detection of poultry viral genes through environmental metagenomics. Bar plots showing the total number of viral genes detected in A) chicken oropharyngeal, B) chicken cloacal, C) duck oropharyngeal, and D) duck cloacal swabs compared to environmental samples at each timepoint. For each environmental sample type, the totals include only those viral genes that have been detected at least once in the corresponding poultry swab type to which it is compared. Thus, each total comprises viral genes recaptured from the corresponding poultry swab and additional viral genes detected only in the environment at that timepoint.

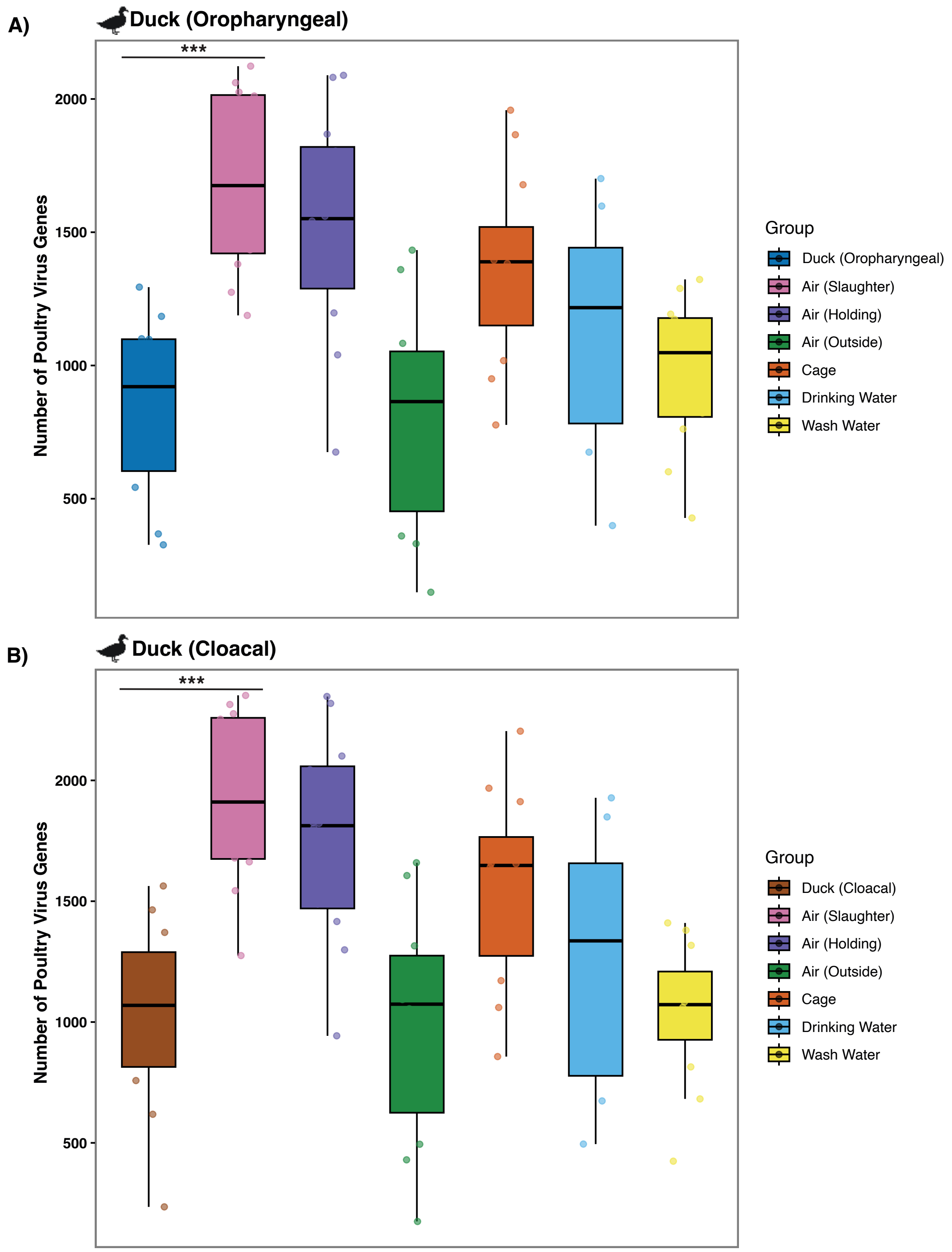

Supplementary Figure 30. Environmental samples detect a higher diversity of poultry virus genes than duck swabs. The total number of poultry virus genes detected in A) duck oropharyngeal and B) duck cloacal swabs is compared to the total number of poultry virus genes in the environment. Statistics were calculated using a Kruskal–Wallis with Dunn's post-hoc test. All P-values obtained were corrected for false discovery rate (FDR) using the Benjamini–Hochberg method. P-values for all pairwise comparisons can be found in Supplementary Tables 31–32. P-values are annotated as follows: $P < 0.05$ *; $P < 0.01$ **; $P < 0.001$ ***.

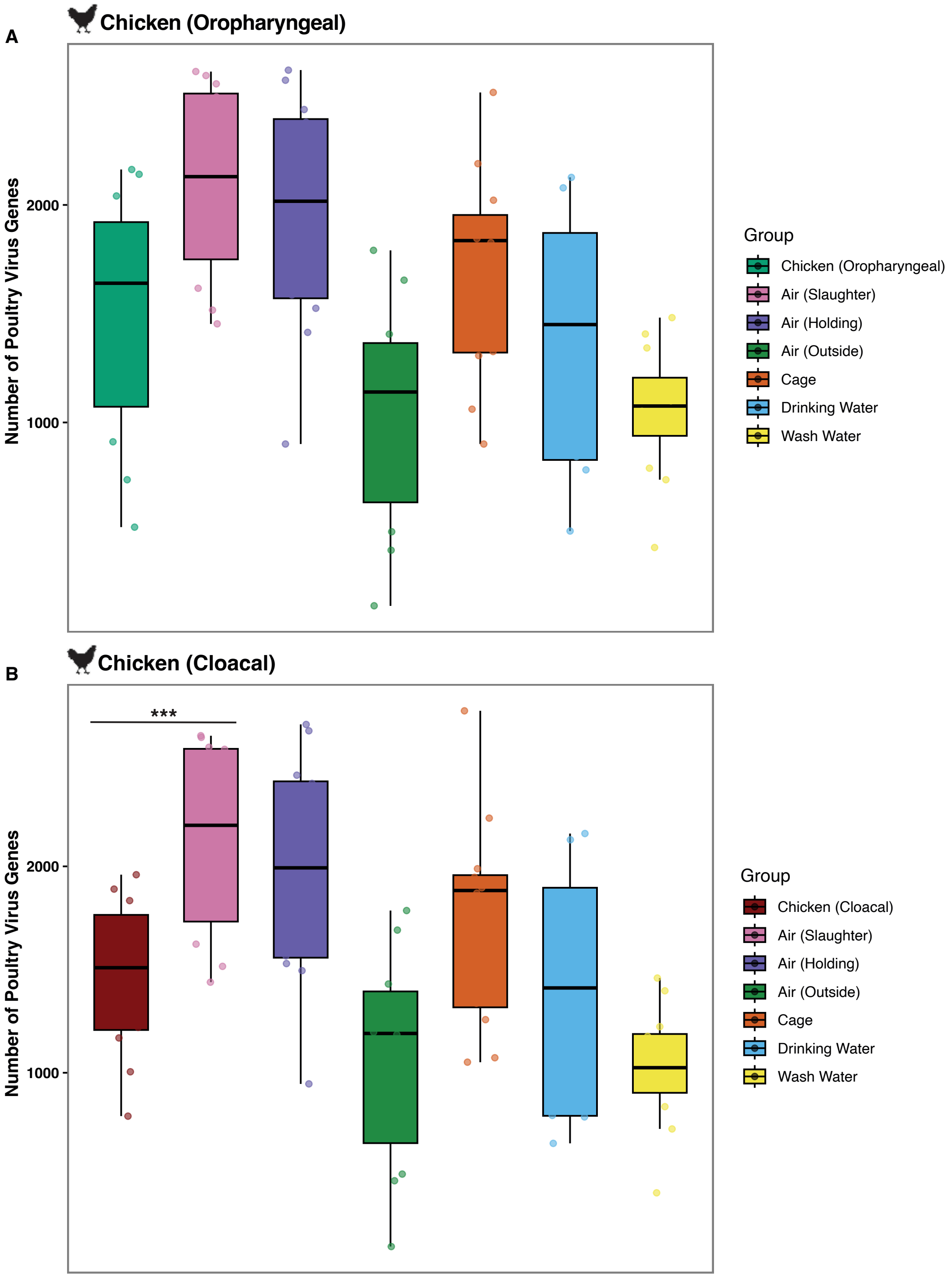

Supplementary Figure 31. Environmental samples detect a higher diversity of poultry virus genes than chicken swabs. The total number of poultry virus genes detected in A) chicken oropharyngeal and B) chicken cloacal swabs is compared to the total number of poultry virus genes in the environment. Statistics were calculated using a Kruskal–Wallis with Dunn's post-hoc test. All P-values obtained were corrected for false discovery rate (FDR) using the Benjamini–Hochberg method. P-values for all pairwise comparisons can be found in Supplementary Tables 33–34. P-values are annotated as follows: $P < 0.05$ *; $P < 0.01$ **; $P < 0.001$ ***.

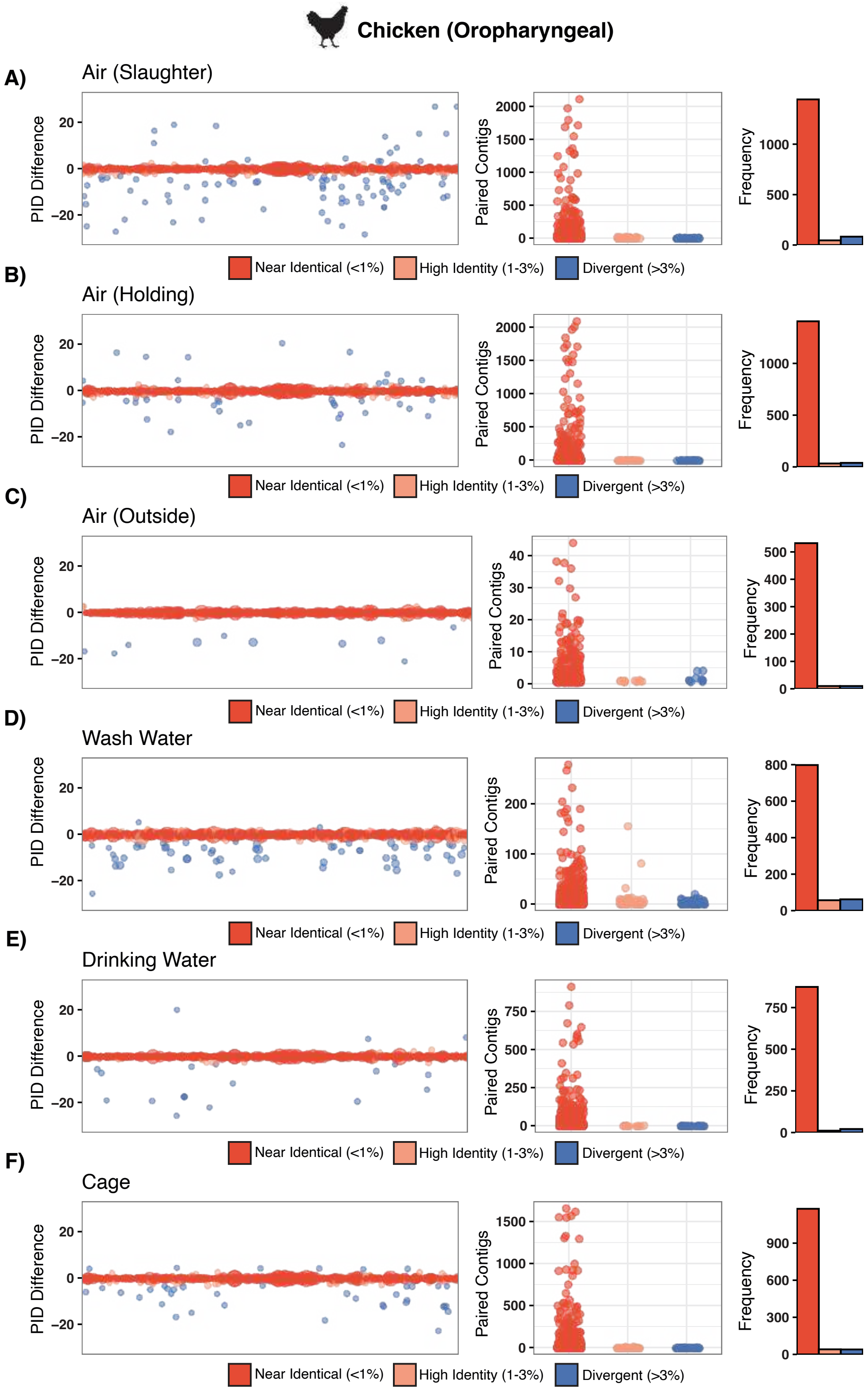

Supplementary Figure 32. The majority of environmental contigs have a high sequence identity to chicken oropharyngeal contigs. Percent identity (PID) to reference genes was estimated for both poultry and environmental contigs with DIAMOND blastx, and the difference between the two PIDs expressed. Chicken oropharyngeal contigs were compared with contigs from (A) air from the slaughter area, (B) air from the holding area, (C) air outside the LBM, (D) carcass wash water, (E) drinking water, and (F) cage swabs. For each environmental sample type, the left panel displays a scatter plot of the difference in PID for every gene, the centre panel summarises the number of poultry–environmental contig pairs across PID thresholds, and the right panel shows the frequency distribution of those pair counts. Statistics were calculated using a Kruskal–Wallis with Dunn's post-hoc test. All P-values obtained were corrected for false discovery rate (FDR) using the Benjamini–Hochberg method.

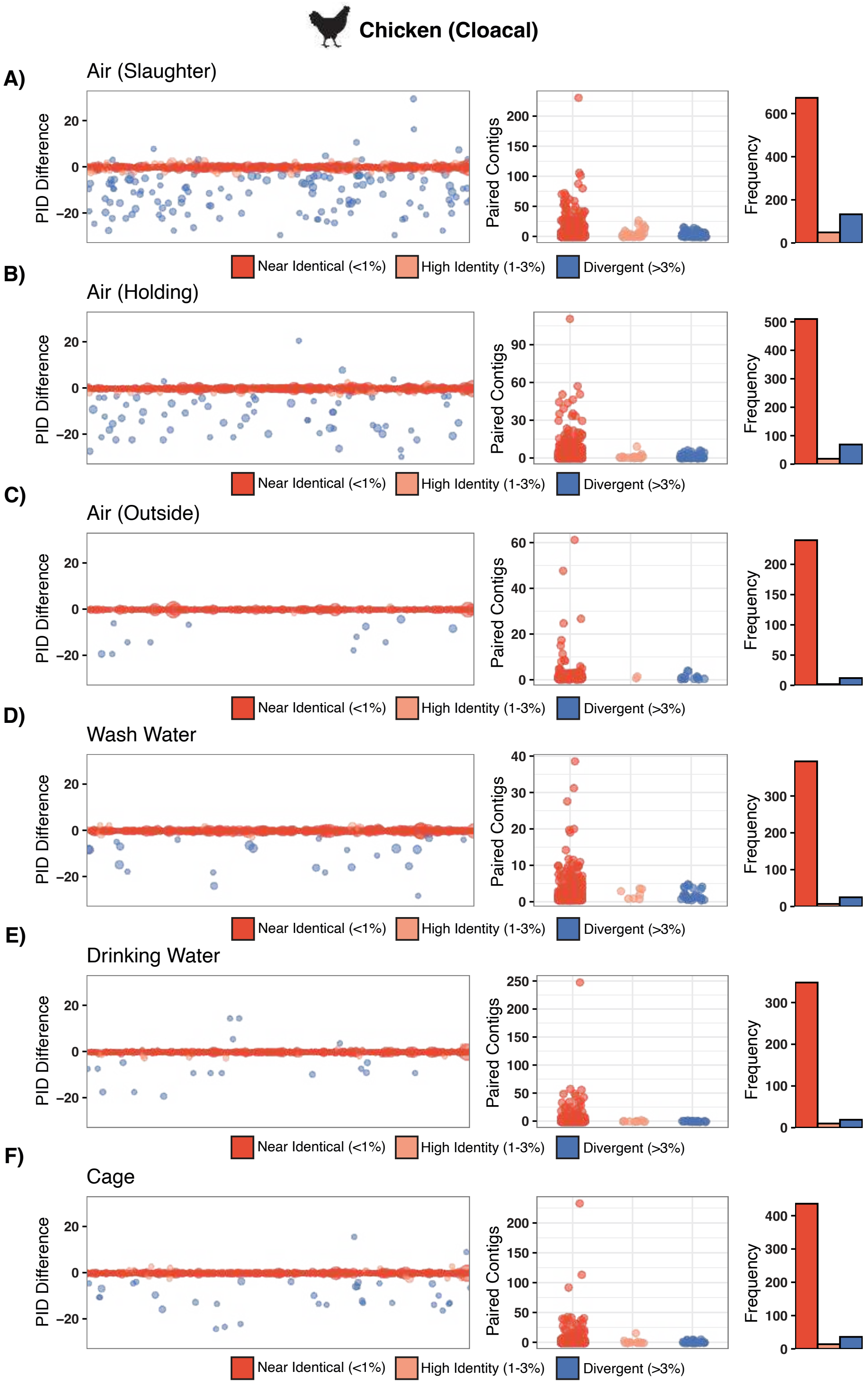

Supplementary Figure 33. The majority of environmental contigs have a high sequence identity to chicken cloacal contigs. Percent identity (PID) to reference genes was estimated for both poultry and environmental contigs with DIAMOND blastx, and the difference between the two PIDs expressed. Chicken cloacal contigs were compared with contigs from (A) air from the slaughter area, (B) air from the holding area, (C) air outside the LBM, (D) carcass wash water, (E) drinking water, and (F) cage swabs. For each environmental sample type, the left panel displays a scatter plot of the difference in PID for every gene, the centre panel summarises the number of poultry–environmental contig pairs across PID thresholds, and the right panel shows the frequency distribution of those pair counts. Statistics were calculated using a Kruskal–Wallis with Dunn’s post-hoc test. All P-values obtained were corrected for false discovery rate (FDR) using the Benjamini–Hochberg method

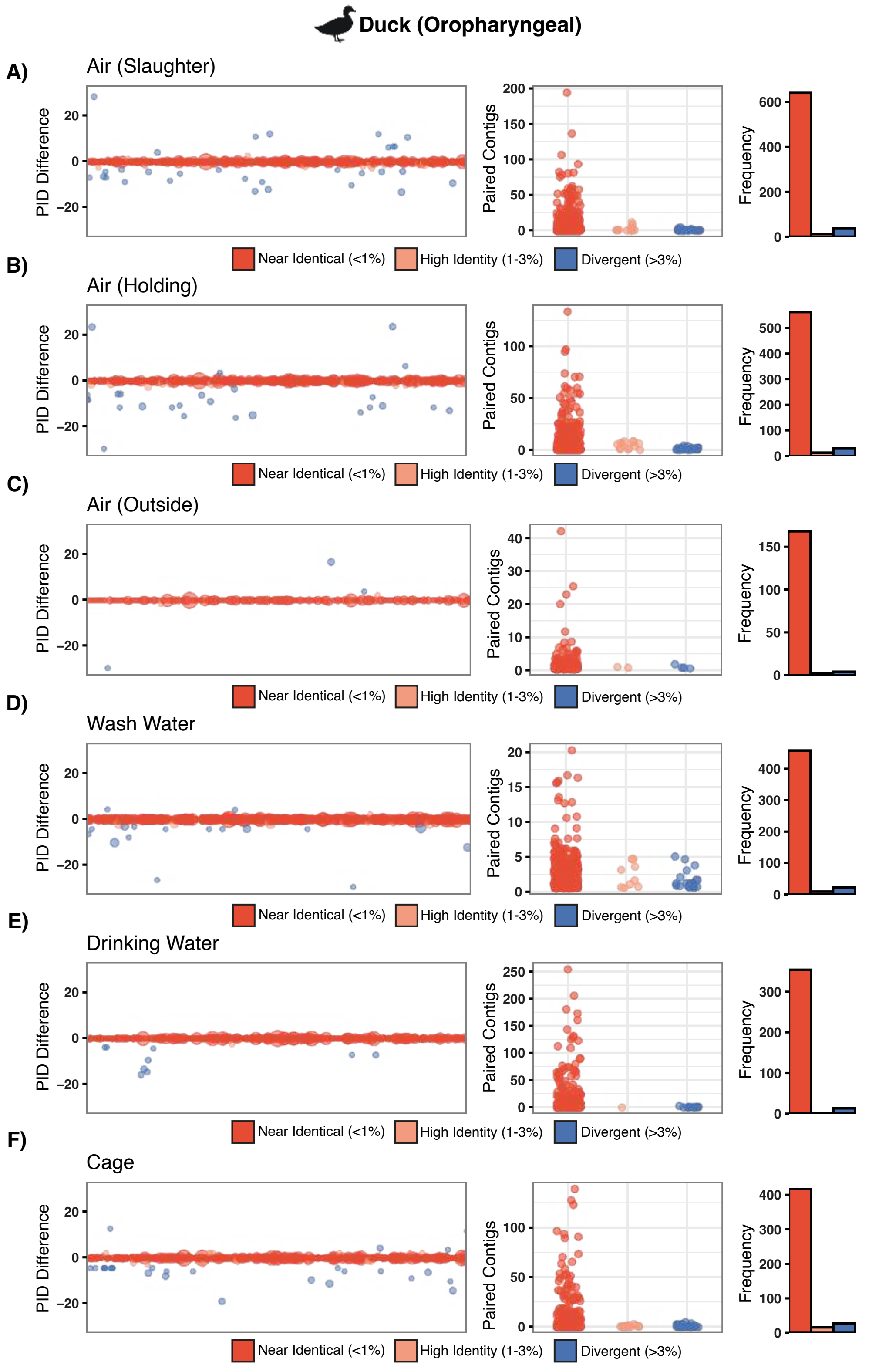

Supplementary Figure 34. The majority of environmental contigs have a high sequence identity to duck oropharyngeal contigs. Percent identity (PID) to reference genes was estimated for both poultry and environmental contigs with DIAMOND blastx, and the difference between the two PIDs expressed. Duck oropharyngeal contigs were compared with contigs from (A) air from the slaughter area, (B) air from the holding area, (C) air outside the LBM, (D) carcass wash water, (E) drinking water, and (F) cage swabs. For each environmental sample type, the left panel displays a scatter plot of the difference in PID for every gene, the centre panel summarises the number of poultry–environmental contig pairs across PID thresholds, and the right panel shows the frequency distribution of those pair counts. Statistics were calculated using a Kruskal–Wallis with Dunn’s post-hoc test.

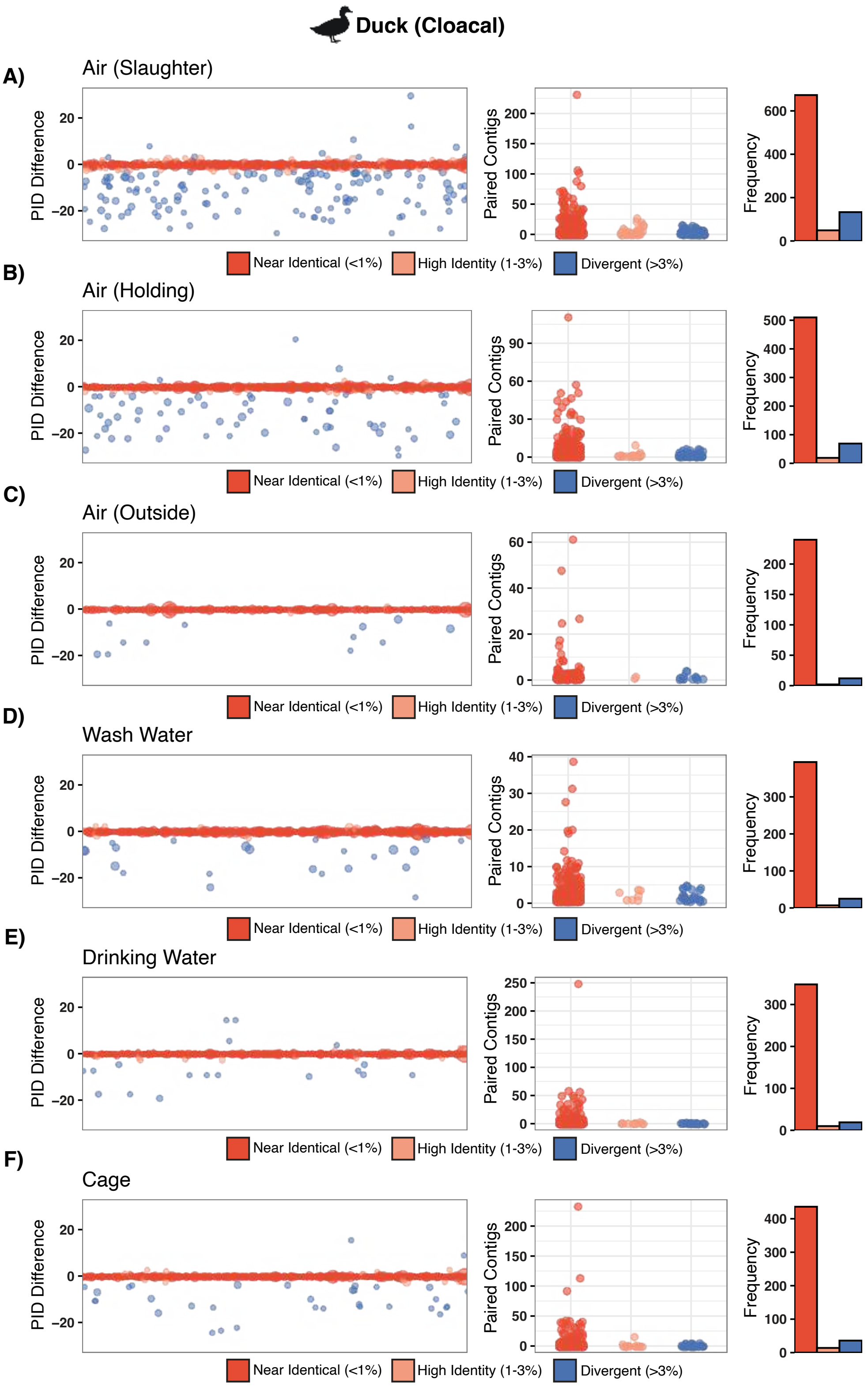

Supplementary Figure 35. The majority of environmental contigs have a high sequence identity to duck cloacal contigs. Percent identity (PID) to reference genes was estimated for both poultry and environmental contigs with DIAMOND blastx, and the difference between the two PIDs expressed. Duck cloacal contigs were compared with contigs from (A) air from the slaughter area, (B) air from the holding area, (C) air outside the LBM, (D) carcass wash water, (E) drinking water, and (F) cage swabs. For each environmental sample type, the left panel displays a scatter plot of the difference in PID for every gene, the centre panel summarises the number of poultry–environmental contig pairs across PID thresholds, and the right panel shows the frequency distribution of those pair counts. Statistics were calculated using a Kruskal–Wallis with Dunn's post-hoc test. All P-values obtained were corrected for false discovery rate (FDR) using the Benjamini–Hochberg method.

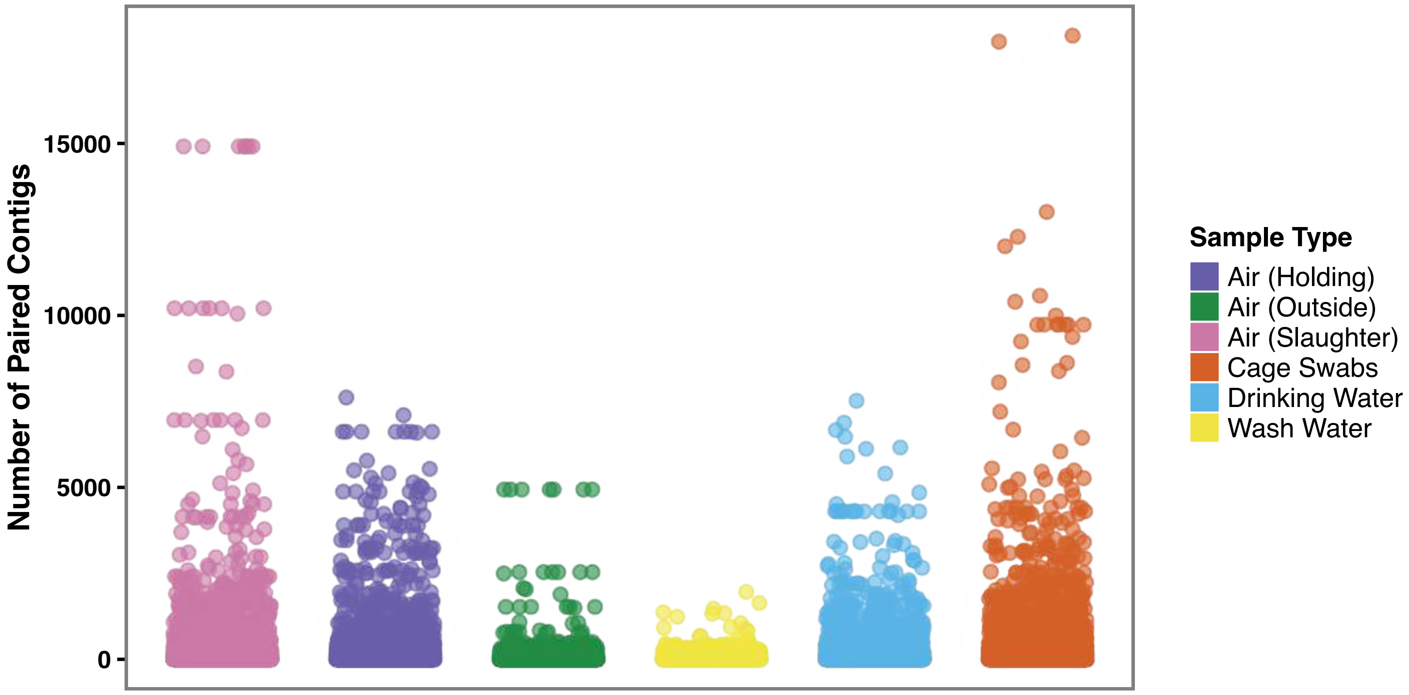

Supplementary Figure 36. Number of poultry environment contig pairs which align to the same virus gene. Summary of the number of environmental contigs in each group which align to the same gene as poultry contigs using DIAMOND blastx. Data from all poultry swab types and sampling occasions are included. Statistics were calculated using a Kruskal–Wallis with Dunn's post-hoc test. All P-values obtained were corrected for false discovery rate (FDR) using the Benjamini–Hochberg method.

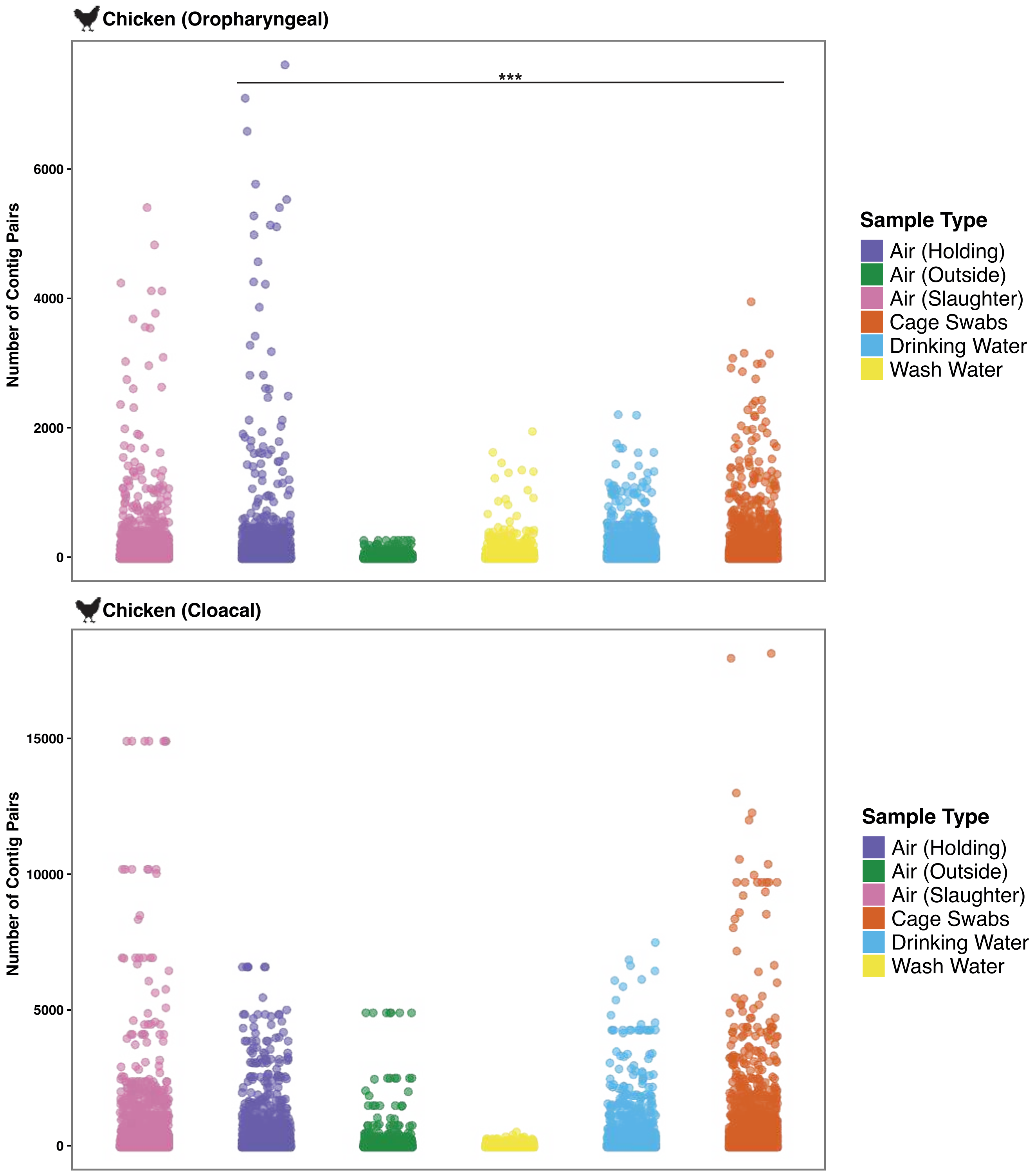

Supplementary Figure 37. Number of chicken swab environment contig pairs which align to the same virus gene. Summary of the number of environmental contigs in each group which align to the same gene as chicken-derived contigs using DIAMOND blastx. Data from all sampling occasions are included. Statistics were calculated using a Kruskal–Wallis with Dunn's post-hoc test. All P-values obtained were corrected for false discovery rate (FDR) using the Benjamini–Hochberg method. P-values for all pairwise comparisons can be found in Supplementary Tables 35–36. P-values are annotated as follows: $P < 0.05$ *; $P < 0.01$ **; $P < 0.001$ ***.

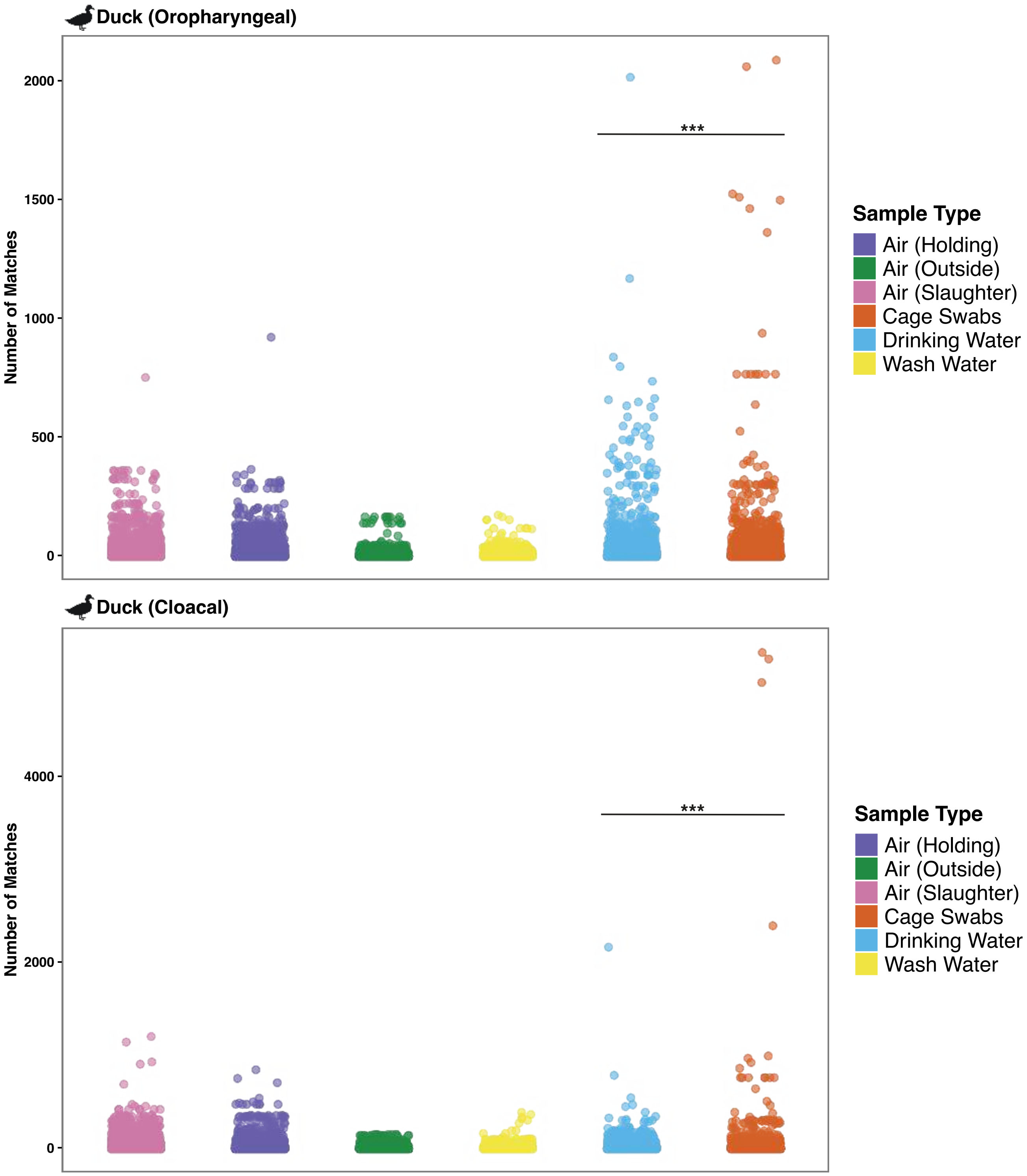

Supplementary Figure 38. Number of duck swab environment contig pairs which align to the same virus gene. Summary of the number of environmental contigs in each group which align to the same gene as duck-derived contigs using DIAMOND blastx. Data from all sampling occasions are included. Statistics were calculated using a Kruskal–Wallis with Dunn's post-hoc test. All P-values obtained were corrected for false discovery rate (FDR) using the Benjamini–Hochberg method. P-values for all pairwise comparisons can be found in Supplementary Data 37–38. P-values are annotated as follows: $P < 0.05$ *; $P < 0.01$ **; $P < 0.001$ ***.

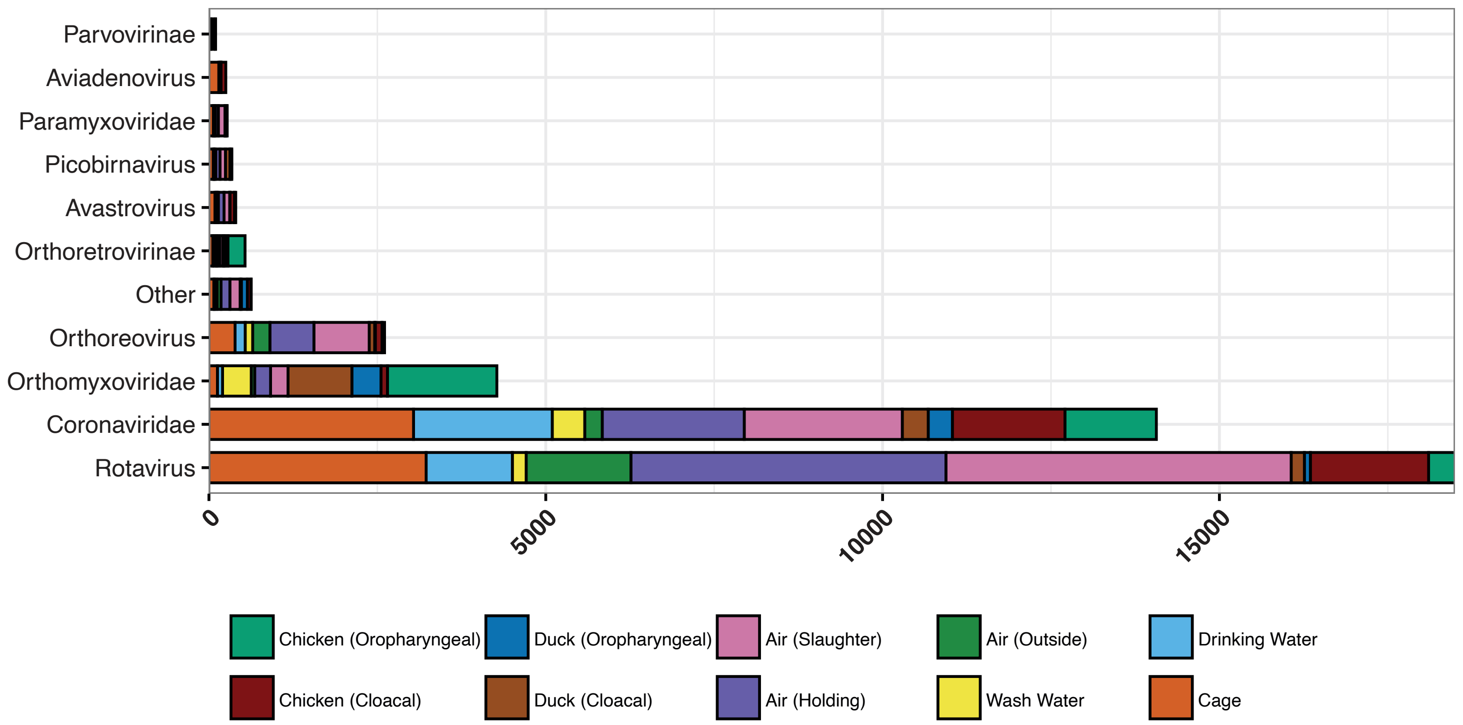

Supplementary Figure 39. A high proportion of poultry environment contig pairs come from *Coronaviridae*, *Rotavirus* and *Orthomyxoviridae*. Stacked bar plot showing the number of matching poultry–environment contig pairs for the top ten virus genera, with each bar segment representing the proportional contribution of each poultry swab type. Data are aggregated across all swab types and sampling occasions..

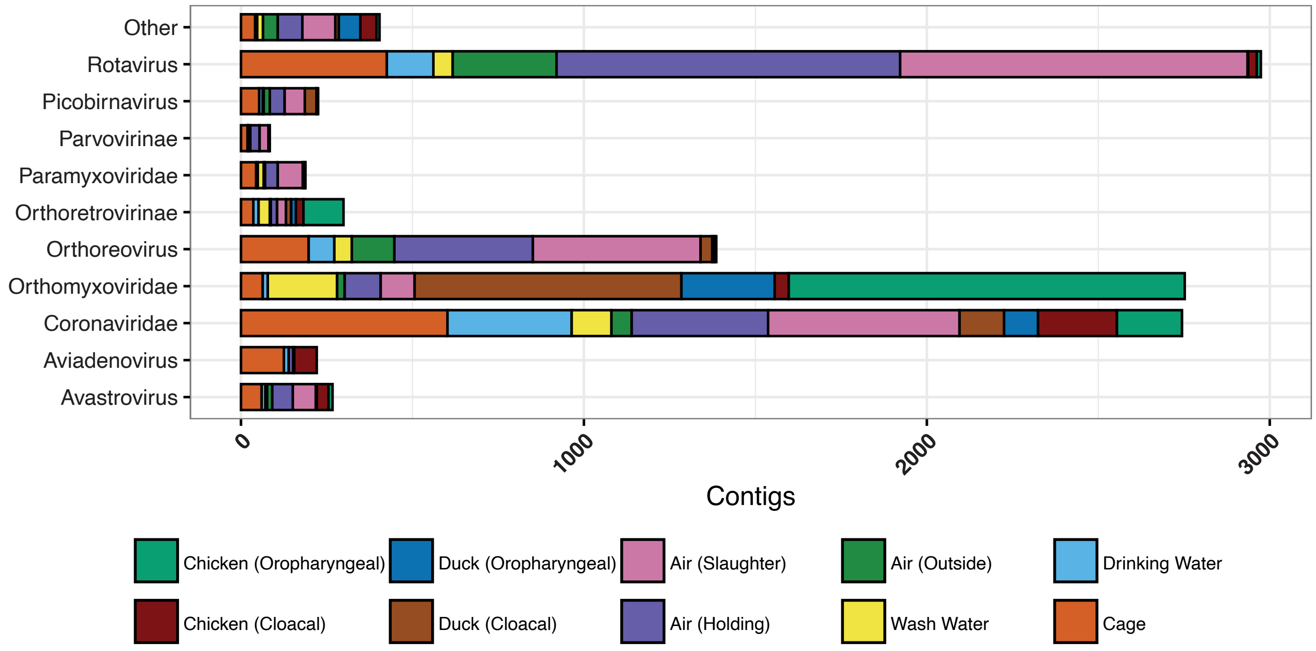

Supplementary Figure 40. Relative proportion of contigs which failed to have a corresponding pair for each of the top ten most abudnant genera. Stacked bar plot showing the number of non-matching poultry and environmental contig pairs for the top ten virus genera, with each bar segment representing the proportional contribution of each poultry swab type. Data are aggregated across all swab types and sampling occasions.

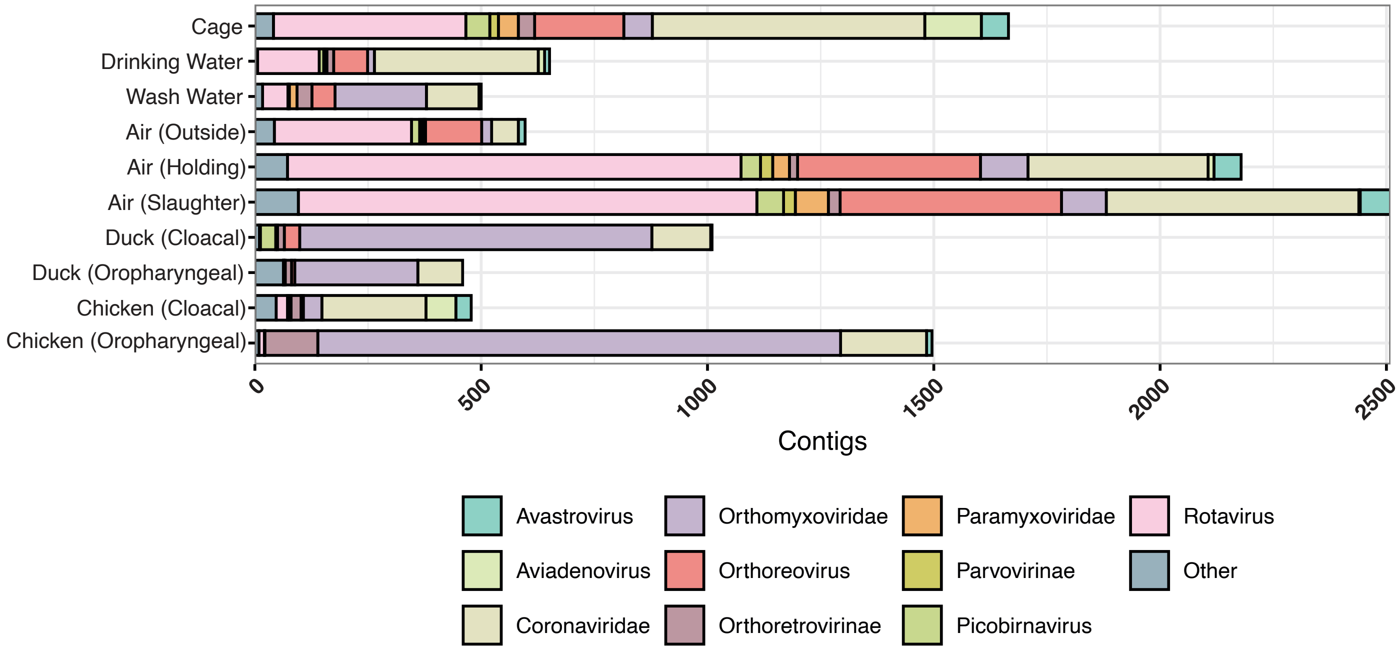

Supplementary Figure 41. A high proportion of poultry environment contig pairs come from *Coronaviridae*, *Rotavirus* and *Orthomyxoviridae*. Stacked bar plot showing the number of non-matching poultry and environmental contigs for each sample type, with each bar segment representing the proportional contribution of each of the top ten virus genera. Data are aggregated across all swab types and sampling occasions.

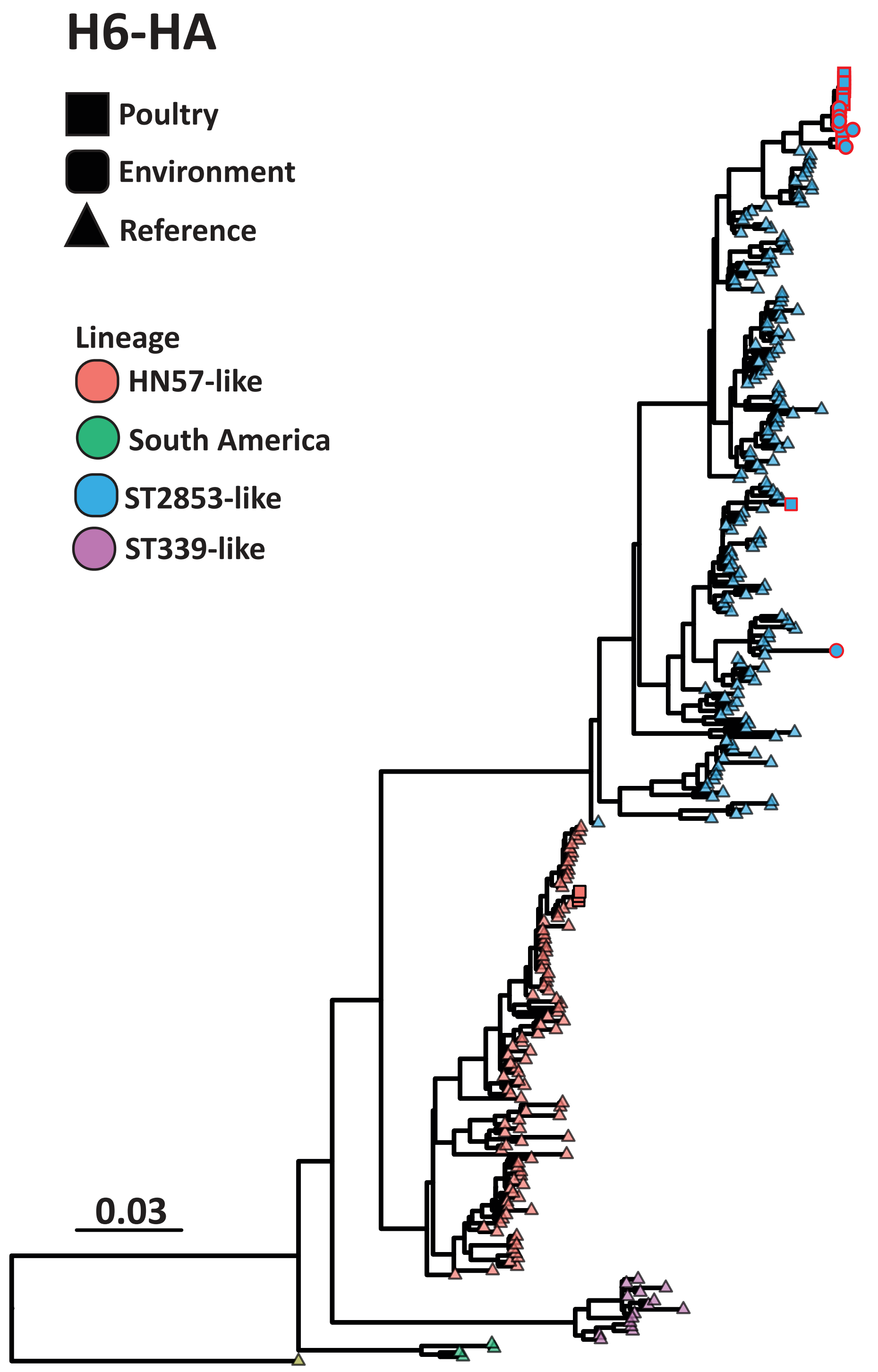

Supplementary Figure 42: HA-H6 sequences identified in the environment and poultry belong to eurasian ST283 clade. Phylogenetic tree showing that environmental sequences cluster closely with those from poultry swabs for HA-H6 (top of the tree). The phylogeny was inferred using 1000 bootstrap replicates, and terminal branches with parental support <70 % were removed, retaining a single connected topology. Circles indicate environmental sequences, squares indicate poultry sequences, and triangles indicate reference sequences from GISAID/NCBI; tips are coloured by clade. The red stroke around the tip highlights new sequences generated in this study.

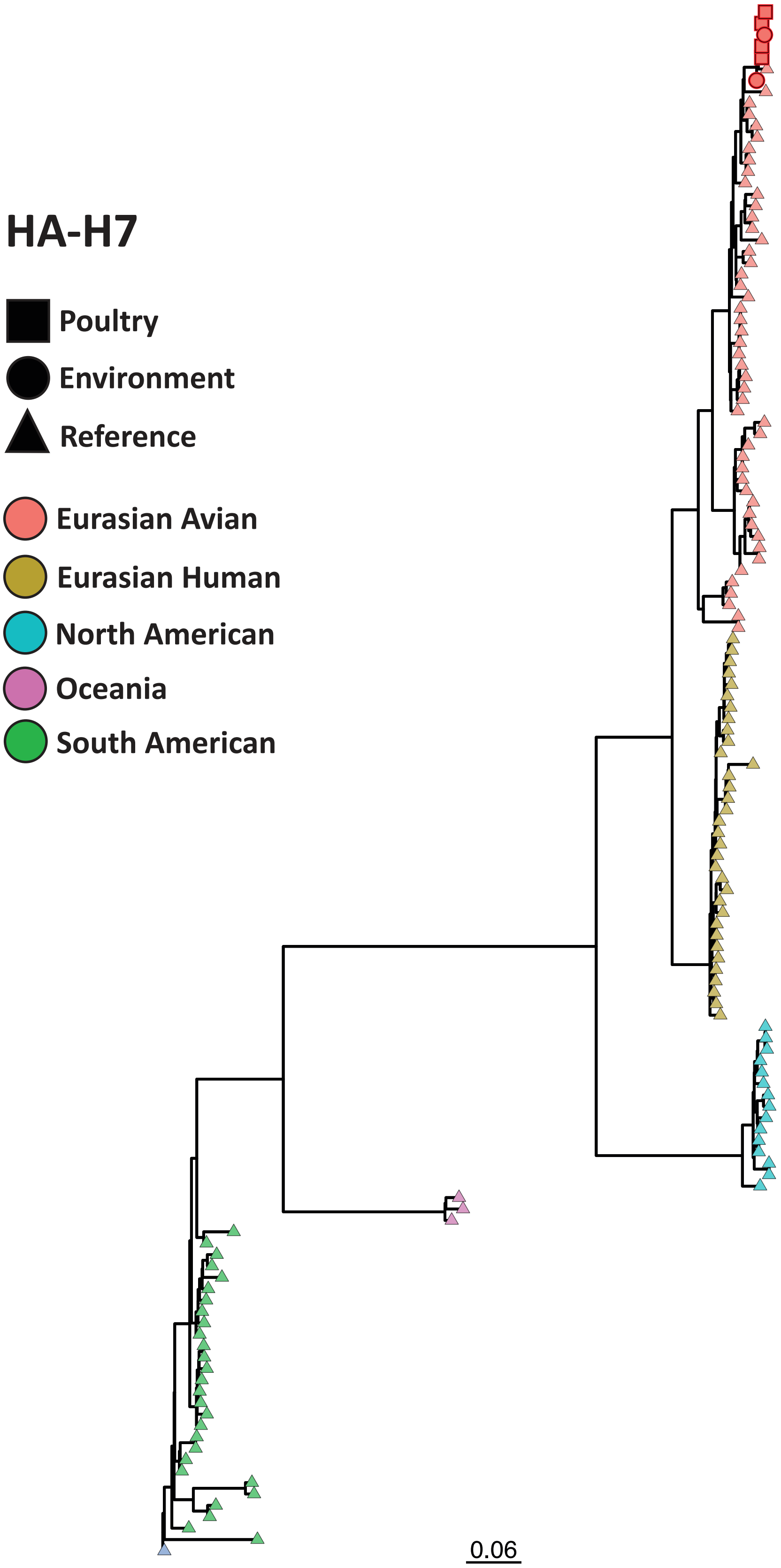

Supplementary Figure 43: HA-H7 sequences identified in the environment and poultry belong to eurasian avian clade. Phylogenetic tree showing that environmental sequences cluster closely with those from poultry swabs for HA-H7 (top of the tree). The phylogeny was inferred using 1000 bootstrap replicates, and terminal branches with parental support <70 % were pruned to retain a single connected topology. Circles represent environmental sequences, squares represent poultry sequences, and triangles indicate reference sequences from GISAID/NCBI; tips are coloured by clade. The red stroke around the tip highlights new sequences generated in this study.
