# Supplementary material for: Environmental metagenomics enhances detection of circulating viruses from live poultry markets in Cambodia
Source: Nat Commun. 2026 Jan 12;17:1525. doi: 10.1038/s41467-025-68245-8 (PMC12891645; doi:10.1038/s41467-025-68245-8)
Supplement: Supplementary file 3 — Description of Additional Supplementary Files [file 41467_2025_68245_MOESM3_ESM.pdf]

File Name: Supplementary Data 1
Description: Number of samples collected for each group at each LBM visit.

File Name: Supplementary Data 2
Description: CZ ID pipeline QC and read trimming summary

File Name: Supplementary Data 3
Description: GenBank protein annotations for DIAMOND blastx results

File Name: Supplementary Data 4
Description: DIAMOND blastx results for each poultry and environmental contig assembled

File Name: Supplementary Data 5
Description: All genome sequences and associated metadata in this dataset are published in GISAID's EpiFlu database.

File Name: Supplementary Data 6
Description: Statistics summary of chicken oropharyngeal virus recapture rates in environmental samples (see Supplementary Fig. 9)

File Name: Supplementary Data 7
Description: Statistics summary of chicken cloacal virus recapture rates in environmental samples (see Supplementary Fig. 9)

File Name: Supplementary Data 8
Description: Statistics summary of duck oropharyngeal virus recapture rates in environmental samples (see Supplementary Fig. 10)

File Name: Supplementary Data 9
Description: Statistics summary of duck cloacal virus recapture rates in environmental samples (see Supplementary Fig. 10)

File Name: Supplementary Data 10
Description: Statistics summary of observed species alpha-diversity between groups (see Fig. 1B)

File Name: Supplementary Data 11
Description: Statistics summary of chao1 alpha-diversity between groups (see Supplementary Fig. 14)

File Name: Supplementary Data 12
Description: Statistics summary of simpson alpha-diversity between groups (see Supplementary Fig. 14)

File Name: Supplementary Data 13
Description: Statistics summary of shannon alpha-diversity between groups (see Supplementary Fig. 14)

File Name: Supplementary Data 14
Description: Statistics summary of comparing the total number of chicken oropharyngeal viruses detected between the groups (see Fig. 1C)

File Name: Supplementary Data 15
Description: Statistics summary of comparing the total number of chicken cloacal viruses detected between the groups (see Fig. 1D)

File Name: Supplementary Data 16
Description: Statistics summary of comparing the total number of duck oropharyngeal viruses detected between the groups (see Fig. 1E)

File Name: Supplementary Data 17
Description: Statistics summary of comparing the total number of duck cloacal viruses detected between the groups (see Fig. 1F)

File Name: Supplementary Data 18
Description: Pairwise PERMANOVA to determine statistically significant differences in virome composition between sample types

File Name: Supplementary Data 19
Description: Statistics summary of comparing the distance from the chicken oropharyngeal median centroid (see Fig. 2C)

File Name: Supplementary Data 20
Description: Statistics summary of comparing the distance from the chicken cloacal median centroid (see Fig. 2D)

File Name: Supplementary Data 21
Description: Statistics summary of comparing the distance from the duck oropharyngeal median centroid (see Fig. 2E)

File Name: Supplementary Data 22
Description: Statistics summary of comparing the distance from the duck oropharyngeal median centroid (see Fig. 2F)

File Name: Supplementary Data 23
Description: P values calculated when comparing the percentage viral contigs in chicken throat swabs that map to ES

File Name: Supplementary Data 24
Description: P values calculated when comparing the percentage viral contigs in chicken cloacal swabs that map to ES

File Name: Supplementary Data 25
Description: P values calculated when comparing the percentage viral contigs in duck throat swabs that map to ES

File Name: Supplementary Data 26
Description: P values calculated when comparing the percentage viral contigs in duck cloacal swabs that map to ES

File Name: Supplementary Data 27
Description: Statistics summary of comparing the number of chicken oropharyngeal virus genes between groups (see Supplementary Fig. 25)

File Name: Supplementary Data 28
Description: Statistics summary of comparing the number of chicken cloacal virus genes between groups (see Supplementary Fig. 25)

File Name: Supplementary Data 29
Description: Statistics summary of comparing the number of duck oropharyngeal virus genes between groups (see Supplementary Fig. 26)

File Name: Supplementary Data 30
Description: Statistics summary of comparing the number of duck cloacal virus genes between groups (see Supplementary Fig. 26)

File Name: Supplementary Data 31
Description: Statistics summary of comparing the total number of duck oropharyngeal virus genes between groups (see Supplementary Fig. 30)

File Name: Supplementary Data 32
Description: Statistics summary of comparing the total number of duck cloacal virus genes between groups (see Supplementary Fig. 30)

File Name: Supplementary Data 33
Description: Statistics summary of comparing the total number of chicken oropharyngeal virus genes between groups (see Supplementary Fig. 31)

File Name: Supplementary Data 34
Description: Statistics summary of comparing the total number of chicken cloacal virus genes between groups (see Supplementary Fig. 31)

File Name: Supplementary Data 35
Description: Statistics summary of comparing the total number of contig pairs which align to chicken oropharyngeal virus genes (see Supplementary Fig. 37)

File Name: Supplementary Data 36
Description: Statistics summary of comparing the total number of contig pairs which align to chicken cloacal virus genes (see Supplementary Fig. 37)

File Name: Supplementary Data 37
Description: Statistics summary of comparing the total number of contig pairs which align to duck oropharyngeal virus genes (see Supplementary Fig. 38)

File Name: Supplementary Data 38
Description: Statistics summary of comparing the total number of contig pairs which align to duck cloacal virus genes (see Supplementary Fig. 38)
