# Supplementary material for: Environmental metagenomics enhances detection of circulating viruses from live poultry markets in Cambodia
Source: Nat Commun. 2026 Jan 12;17:1525. doi: 10.1038/s41467-025-68245-8 (PMC12891645; doi:10.1038/s41467-025-68245-8)
Supplement: Supplementary file 8 — Supplementary Data 5 [file 41467_2025_68245_MOESM8_ESM.pdf]

SUPPLEMENTAL TABLE

Data Availability

GISAID Identifier: EPI_SET_250703up
DOI: https://doi.org/10.55876/gis8.250703up

All genome sequences and associated metadata in this dataset are published in GISAID's EpiFlu database. To view the contributors of each individual sequence with details such as accession number, Virus name, Collection date, Originating Lab and Submitting Lab and the list of Authors, visit EPI_SET_250703up

Data Snapshot

EPI_SET_250703up is composed of 2531 individual viruses.
The collection dates range from 2014-01-01 to 2024-05-17;
Data were collected in 91 countries and territories.
