# Supplementary material for: Environmental metagenomics enhances detection of circulating viruses from live poultry markets in Cambodia
Source: Nat Commun. 2026 Jan 12;17:1525. doi: 10.1038/s41467-025-68245-8 (PMC12891645; doi:10.1038/s41467-025-68245-8)
Supplement: Supplementary file 10 — Reporting Summary [file 41467_2025_68245_MOESM10_ESM.pdf]

nature portfolio

Corresponding author(s): Erik A. Karlsson

Last updated by author(s): 20/1/25

Reporting Summary

Nature Portfolio wishes to improve the reproducibility of the work that we publish. This form provides structure for consistency and transparency in reporting. For further information on Nature Portfolio policies, see our Editorial Policies and the Editorial Policy Checklist.

Statistics

For all statistical analyses, confirm that the following items are present in the figure legend, table legend, main text, or Methods section.

n/a	Confirmed	
☐	☒	The exact sample size (n) for each experimental group/condition, given as a discrete number and unit of measurement
☐	☒	A statement on whether measurements were taken from distinct samples or whether the same sample was measured repeatedly
☐	☒	The statistical test(s) used AND whether they are one- or two-sided<br>*Only common tests should be described solely by name; describe more complex techniques in the Methods section.*
☒	☐	A description of all covariates tested
☐	☒	A description of any assumptions or corrections, such as tests of normality and adjustment for multiple comparisons
☐	☒	A full description of the statistical parameters including central tendency (e.g. means) or other basic estimates (e.g. regression coefficient) AND variation (e.g. standard deviation) or associated estimates of uncertainty (e.g. confidence intervals)
☐	☒	For null hypothesis testing, the test statistic (e.g. F, t, r) with confidence intervals, effect sizes, degrees of freedom and P value noted<br>*Give P values as exact values whenever suitable.*
☒	☐	For Bayesian analysis, information on the choice of priors and Markov chain Monte Carlo settings
☒	☐	For hierarchical and complex designs, identification of the appropriate level for tests and full reporting of outcomes
☒	☐	Estimates of effect sizes (e.g. Cohen's d, Pearson's r), indicating how they were calculated

Our web collection on statistics for biologists contains articles on many of the points above.

Software and code

Policy information about availability of computer code

Data collection	*Provide a description of all commercial, open source and custom code used to collect the data in this study, specifying the version used OR state that no software was used.*
Data analysis	Code and associated data can be found at https://github.com/PeterCx/air-sampling-zoonotic-virus.

For manuscripts utilizing custom algorithms or software that are central to the research but not yet described in published literature, software must be made available to editors and reviewers. We strongly encourage code deposition in a community repository (e.g. GitHub). See the Nature Portfolio guidelines for submitting code & software for further information.

Data

Policy information about availability of data

All manuscripts must include a data availability statement. This statement should provide the following information, where applicable:

- Accession codes, unique identifiers, or web links for publicly available datasets
- A description of any restrictions on data availability
- For clinical datasets or third party data, please ensure that the statement adheres to our policy

Raw sequencing data used in this study are publicly available under accession number PRJEB83776. This is due to be released in August 2025 at a time when the paper should be published. Detailed metadata associated with the study are also accessible at https://github.com/PeterCx/air-sampling-zoonotic-virus to ensure transparency and reproducibility.

Research involving human participants, their data, or biological material

Policy information about studies with human participants or human data. See also policy information about sex, gender (identity/presentation), and sexual orientation and race, ethnicity and racism.

Reporting on sex and gender	*Use the terms sex (biological attribute) and gender (shaped by social and cultural circumstances) carefully in order to avoid confusing both terms. Indicate if findings apply to only one sex or gender; describe whether sex and gender were considered in study design; whether sex and/or gender was determined based on self-reporting or assigned and methods used. Provide in the source data disaggregated sex and gender data, where this information has been collected, and if consent has been obtained for sharing of individual-level data; provide overall numbers in this Reporting Summary. Please state if this information has not been collected. Report sex- and gender-based analyses where performed, justify reasons for lack of sex- and gender-based analysis.*
Reporting on race, ethnicity, or other socially relevant groupings	*Please specify the socially constructed or socially relevant categorization variable(s) used in your manuscript and explain why they were used. Please note that such variables should not be used as proxies for other socially constructed/relevant variables (for example, race or ethnicity should not be used as a proxy for socioeconomic status). Provide clear definitions of the relevant terms used, how they were provided (by the participants/respondents, the researchers, or third parties), and the method(s) used to classify people into the different categories (e.g. self-report, census or administrative data, social media data, etc.) Please provide details about how you controlled for confounding variables in your analyses.*
Population characteristics	*Describe the covariate-relevant population characteristics of the human research participants (e.g. age, genotypic information, past and current diagnosis and treatment categories). If you filled out the behavioural & social sciences study design questions and have nothing to add here, write "See above."*
Recruitment	*Describe how participants were recruited. Outline any potential self-selection bias or other biases that may be present and how these are likely to impact results.*
Ethics oversight	*Identify the organization(s) that approved the study protocol.*

Note that full information on the approval of the study protocol must also be provided in the manuscript.

Field-specific reporting

Please select the one below that is the best fit for your research. If you are not sure, read the appropriate sections before making your selection.

☐ Life sciences ☐ Behavioural & social sciences ☒ Ecological, evolutionary & environmental sciences

For a reference copy of the document with all sections, see nature.com/documents/nr-reporting-summary-flat.pdf

Ecological, evolutionary & environmental sciences study design

All studies must disclose on these points even when the disclosure is negative.

Study description	In this study, we assessed the performance of metagenomics on environmental samples (ES) compared to traditional poultry swabs for detecting avian viral pathogens in LBMs in Cambodia. ES, including air, cage swabs, and carcass wash water, were collected alongside throat and cloacal swabs from domestic chickens and ducks across twelve sampling visits in two LBMs over a 15-month period. Viral nucleic acids were extracted and sequenced using a capture probe-based metagenomics approach.
Research sample	Individual animal samples consisted of oropharyngeal and cloacal swabs collected from an domestic ducks and chickens. These were combined into pools of ten individual cloacal or oropharyngeal swabs for sample processing, with ducks and chickens pooled separately. Environmental samples comprised air, carcass wash water, cage swabs, and drinking water.
Sampling strategy	Air samples were collected for three hours at three locations at each market (the slaughter area, the poultry holding/storage area, and a "control area" - either the food storage area located in a separate building or >50m away from the LBM) using AerosolSenseTM Samplers (Thermo Fisher Scientific). Air was sampled through an omnidirectional inlet at a rate of 200 L/min. Collected material was eluted from each air sampler by rinsing and vortexing the collection matrix in 2 mL of viral transport medium. Carcass wash water (50 mL) used to wash poultry carcasses after slaughter and de-feathering processes was collected from the slaughter area. Cage swabs were collected from selected cages in the poultry holding area of the market. Where available, 2 mL of poultry drinking water from inside poultry cages was collected.
Data collection	RNA was extracted from each sample pool using the Direct-zol RNA Miniprep Kit (Zymo Research, California, USA) according to the manufacturer's protocol and eluted in 50uL of nuclease-free water. Sequencing libraries were generated using the Twist Library Preparation Enzymatic Fragmentation (EF) kit 2.0 (Twist Bioscience, San Francisco, California, USA) and the Twist Comprehensive Viral Research Panel (CVRP), according to the manufacturer's protocol (Twist Total Nucleic Acids Library Preparation EF Kit 2.0 for Viral Pathogen Detection and Characterization Protocol and Twist Target Enrichment Standard Hybridization v1 protocol). Briefly, extracted RNA was converted to cDNA using the ProtoScript II First Strand cDNA Synthesis kit (New England Biolabs, Ipswich, Massachusetts, USA) and Random Primer 6 (S1230S) from New England Biolabs (NEB). The NEB Next Ultra II Non-Directional RNA second Strand Synthesis kit (E6111S) was subsequently used to convert the single-stranded cDNA to double stranded DNA (dsDNA). Illumina TruSeq-compatible libraries were generated using the Twist Library Preparation EF kit 2.0 and combined into pools of six libraries. The pooled libraries were enriched by hybridization capture using the Twist Comprehensive Viral Research Panel (CVRP).

	Following enrichment, libraries were pooled in equimolar ratios, diluted and denatured prior to sequencing according to the standard MiSeq System Denature and Dilute Libraries Guide (Document # 15039740v10). Sequencing was performed Finally, the library was sequenced to generate paired-end 75bp reads using a MiSeq V3 reagent kit (Illumina, MS-102-3001) on the Illumina MiSeq System.
Timing and spatial scale	Between January 2022 and April 2023 samples were collected on 12 occasions in Cambodia at Orussey market, Phnom Penh province and Daun Keo market, Takeo province. The exact dates are as follows: 27-01-2022, 17-03-2022, 12-04-2022, 22-04-2022, 21-12-2022, 21-12-2022, 23-12-2022, 19-01-2023, 07-03-2023, 10-03-2023, 12-04-2023.
Data exclusions	No data was excluded from the analysis
Reproducibility	No formal attempts to repeat the experiments described in this study have been made. While reproducibility was not directly assessed, detailed methodologies, including all reagents, experimental protocols, and data processing workflows, are provided to ensure that independent researchers can replicate the findings if desired.
Randomization	Random allocation of samples was not applicable to this study, as the objective was to collect and analyze environmental and biological samples from specific locations (markets) and sources (domestic ducks and chickens). Sampling was performed systematically based on predefined locations within the markets (slaughter area, poultry holding area, and control area) and specific types of samples (oropharyngeal and cloacal swabs, air, carcass wash water, cage swabs, and drinking water).<br>To control for potential covariates, samples were processed and analyzed separately for each species (ducks and chickens) and sample type, ensuring consistent and unbiased handling across all groups.
Blinding	Blinding was not implemented during data acquisition or analysis in this study, as the nature of the research involved environmental and biological sampling from specific locations and sources, which were inherently identifiable. The primary objective was to systematically assess and compare samples from defined market zones, species (ducks and chickens), and sample types, making blinding irrelevant to the study's design. To minimize potential bias, standardized protocols were followed for sample collection, processing, and analysis, ensuring consistency across all procedures.

Did the study involve field work? ☒ Yes ☐ No

Field work, collection and transport

Field conditions	Between January 2022 and April 2023 samples were collected on 12 occasions in Cambodia at Orussey market, Phnom Penh province and Daun Keo market, Takeo province. Samples were collected indoors inside the market.
Location	Between January 2022 and April 2023 samples were collected on 12 occasions in Cambodia at Orussey market, Phnom Penh province and Daun Keo market, Takeo province.
Access & import/export	All sample collections conducted for this study adhered to ethical guidelines and were carried out in collaboration with the National Animal Health and Production Institute. The collections were conducted under protocols approved by the National Ethics Committee for Health Research (NECHR) under the Ministry of Health, Cambodia, with approval numbers NECHR013, NECHR143, NECHR149, and NECHR320 approved in 2021 and 2022. Cambodia does not have a specific animal ethics review board; however, further informal approvals for animal sample collections are given by the General Directorate of Animal Health and Production under the Ministry of Agriculture, Forestry and Fisheries.
Disturbance	NA

Reporting for specific materials, systems and methods

We require information from authors about some types of materials, experimental systems and methods used in many studies. Here, indicate whether each material, system or method listed is relevant to your study. If you are not sure if a list item applies to your research, read the appropriate section before selecting a response.

Materials & experimental systems

n/a	Involved in the study
☒	☐ Antibodies
☒	☐ Eukaryotic cell lines
☒	☐ Palaeontology and archaeology
☐	☒ Animals and other organisms
☒	☐ Clinical data
☒	☐ Dual use research of concern
☒	☐ Plants

Methods

n/a	Involved in the study
☒	☐ ChIP-seq
☒	☐ Flow cytometry
☒	☐ MRI-based neuroimaging

Animals and other research organisms

Policy information about studies involving animals; ARRIVE guidelines recommended for reporting animal research, and Sex and Gender in Research

Laboratory animals	Did not involve lab animals
Wild animals	Did not involve wild animals
Reporting on sex	NA
Field-collected samples	NA
Ethics oversight	All sample collections conducted for this study adhered to ethical guidelines and were carried out in collaboration with the National Animal Health and Production Institute. The collections were conducted under protocols approved by the National Ethics Committee for Health Research (NECHR) under the Ministry of Health, Cambodia, with approval numbers NECHR013, NECHR143, NECHR149, and NECHR320 approved in 2021 and 2022. Cambodia does not have a specific animal ethics review board; however, further informal approvals for animal sample collections are given by the General Directorate of Animal Health and Production under the Ministry of Agriculture, Forestry and Fisheries.

Note that full information on the approval of the study protocol must also be provided in the manuscript.

Plants

Seed stocks	*Report on the source of all seed stocks or other plant material used. If applicable, state the seed stock centre and catalogue number. If plant specimens were collected from the field, describe the collection location, date and sampling procedures.*
Novel plant genotypes	*Describe the methods by which all novel plant genotypes were produced. This includes those generated by transgenic approaches, gene editing, chemical/radiation-based mutagenesis and hybridization. For transgenic lines, describe the transformation method, the number of independent lines analyzed and the generation upon which experiments were performed. For gene-edited lines, describe the editor used, the endogenous sequence targeted for editing, the targeting guide RNA sequence (if applicable) and how the editor was applied.*
Authentication	*Describe any authentication procedures for each seed stock used or novel genotype generated. Describe any experiments used to assess the effect of a mutation and, where applicable, how potential secondary effects (e.g. second site T-DNA insertions, mosiacism, off-target gene editing) were examined.*
